# Ensemble modeling highlights importance of understanding parasite-host behavior in preclinical antimalarial drug development

**DOI:** 10.1038/s41598-020-61304-8

**Published:** 2020-03-10

**Authors:** Lydia Burgert, Matthias Rottmann, Sergio Wittlin, Nathalie Gobeau, Andreas Krause, Jasper Dingemanse, Jörg J. Möhrle, Melissa A. Penny

**Affiliations:** 1 Swiss Tropical and Public Health Institute, Basel, Switzerland; 2 University of Basel, Basel, Switzerland; 3 Medicines for Malaria Venture, Geneva, Switzerland; 4 Idorsia Pharmaceuticals Ltd, Clinical Pharmacology, Allschwil, Switzerland

**Keywords:** Computational models, Malaria

## Abstract

Emerging drug resistance and high-attrition rates in early and late stage drug development necessitate accelerated development of antimalarial compounds. However, systematic and meaningful translation of drug efficacy and host-parasite dynamics between preclinical testing stages is missing. We developed an ensemble of mathematical within-host parasite growth and antimalarial action models, fitted to extensive data from four antimalarials with different modes of action, to assess host-parasite interactions in two preclinical drug testing systems of murine parasite *P. berghei* in mice, and human parasite *P. falciparum* in immune-deficient mice. We find properties of the host-parasite system, namely resource availability, parasite maturation and virulence, drive *P. berghei* dynamics and drug efficacy, whereas experimental constraints primarily influence *P. falciparum* infection and drug efficacy. Furthermore, uninvestigated parasite behavior such as dormancy influences parasite recrudescence following non-curative treatment and requires further investigation. Taken together, host-parasite interactions should be considered for meaningful translation of pharmacodynamic properties between murine systems and for predicting human efficacious treatment.

## Introduction

Scale-up of vector control and treatment strategies have led to large reductions in *Plasmodium falciparum* malaria prevalence and clinical cases over the last decade^1^. However, malaria remains a major cause of morbidity and mortality worldwide and recent successes are challenged by emerging resistance against several recommended first line treatments of artemisinin combination therapy^2,3^. Although the current pipeline for new antimalarials is healthy; late stage drug attrition in antimalarial development and the need to develop combination therapies necessitates a continued search for new compounds^4^. Host-parasite dynamics and their influence on treatment results are important to consider throughout drug development to understand and interpret observed drug efficacy. Coupled with data, mechanistic modeling and simulation enables exploration of these host-parasite interactions along the preclinical development pathway. Such models facilitate translation from preclinical murine systems to clinical use, and thus potentially reduce time and costs to develop new antimalarial treatments.

In preclinical antimalarial development stages, murine systems of malaria infection are employed to evaluate drug pharmacokinetics (PK), drug effects (pharmacodynamics), efficacious exposure, and to inform human dose prediction. Pharmacodynamic (PD) measures of evaluation include parasite reduction compared to a control group, index numbers of drug efficacy such as concentrations inhibiting growth or resulting in a certain level of parasiticidal activity, and parasite recrudescence behavior following non-curative treatment^5–7^.

Two murine systems are commonly employed to investigate *in vivo* blood-stage efficacy of orally administered antimalarials; infection of normal mice with the *P. berghei* ANKA strain^8^ and infection of immunodeficient NOD ^scidIL-2R′ c−/−^ (SCID) mice with *P. falciparum*^9–11^. The murine malaria parasite *P. berghei* causes severe, ultimately deadly malaria in mice while exhibiting similar parasite morphology and developmental characteristics observed in human malaria infection^7,12^. SCID mice engrafted with human erythrocytes (RBCs) are able to support infection with *P. falciparum*, providing the opportunity to investigate the efficacy of compounds against the human parasite *in vivo*^10,11^. The main difference between the two parasite species is the length of the intra-erythrocytic life cycle being approximately 24 h for *P. berghei* and approximately 48 h for *P. falciparum*^7^. While the *P. berghei* murine system is used to test crude efficacy of blood-stage antimalarial drugs in shorter experiments, murine infection with *P. falciparum* is employed in longer experiments investigating the course of infection and parasite recrudescence behavior. Recently the SCID mouse system has been utilized to facilitate translation of results between mice and humans^9^, including testing of drug combinations, and to avoid issues where potentially active compounds against *P. falciparum* are not active against *P. berghei* due to enzymatic differences between the parasites^13^.

Mechanistic mathematical parasite growth models inform the drug development process by combining information on within-host behavior of the parasite, the host itself, and the treatment^14,15^. Several within-host models that include descriptions of the asexual blood-stage parasite life cycle and host properties have been developed for preclinical^16–19^ and clinical development stages^14,20–22^. However, modeling is not used to systematically compare potential consequences of host-parasite interactions in different host-parasite systems and to investigate their impact on drug treatment outcomes and decisions during antimalarial development. Comparing performance of models capturing different aspects of biology can indicate importance of those aspects, or point to knowledge gaps.

We report an ensemble of mechanistic within-host parasite growth and antimalarial action models that are combined into a modeling workflow that handles data management, model development, parameterization, and simulation for the analysis of antimalarial drugs in murine experimental systems. The models are based on previously described parasite characteristics such as erythropoiesis, parasite growth, erythrocyte and parasite clearance, and changes in parasite characteristics over the course of infection^23^. Model selection is based on their potential relevance for assessing drug efficacy in preclinical antimalarial development. Our ensemble therefore highlights the diversity of potential parasite-host dynamics and the consequential influence on experimental insights and drug evaluation in the space of limited data resolution of the parasite life cycle. Parameterization was conducted using multiple control and treatment experiments of four antimalarials with different modes of action. We evaluated the models based on their ability to describe laboratory data and to account for the biological and experimental background to understand parasite dynamics relevant for treatment effects. The workflow enables the analysis of *in vivo* drug efficacy against *P. berghei* and *P. falciparum* and thus facilitates comparison of results between laboratories. To the best of our knowledge, this is the first study to systematically investigate host-parasite interactions, antimalarial action, and drug effects across murine experimental systems, laboratories, and drugs from different drug classes. This analysis provides insights into antimalarial efficacy predictions, highlights processes of host-parasite interaction relevant to malaria in humans and informs on the advantages and disadvantages of each preclinical system.

## Results

### Data

The following drugs, for which data on both murine systems was available, were used for analysis: ACT-451840^24–26^, chloroquine (CQ)^27^, MMV390048^27,28^ and OZ439 (INN: artefenomel)^29,30^ (Supplementary Table S1). Data from 43 experiments containing information about *P. berghei* in Naval Medical Research Institute (NMRI) mice and 32 experiments containing information about *P. falciparum* in SCID mice before and after treatment were analyzed. Each experiment involved 2–5 control mice and 2–10 mice per dose. An overview of the data used can be found in the Supplementary Table S2. Parasite density data in the form of percentage of infected RBCs was used for model parameterization. In SCID mice *models f* and *g*, hematocrit (percentage of human RBCs) was used as additional information.

### Models of parasite growth

**Figure 1:**
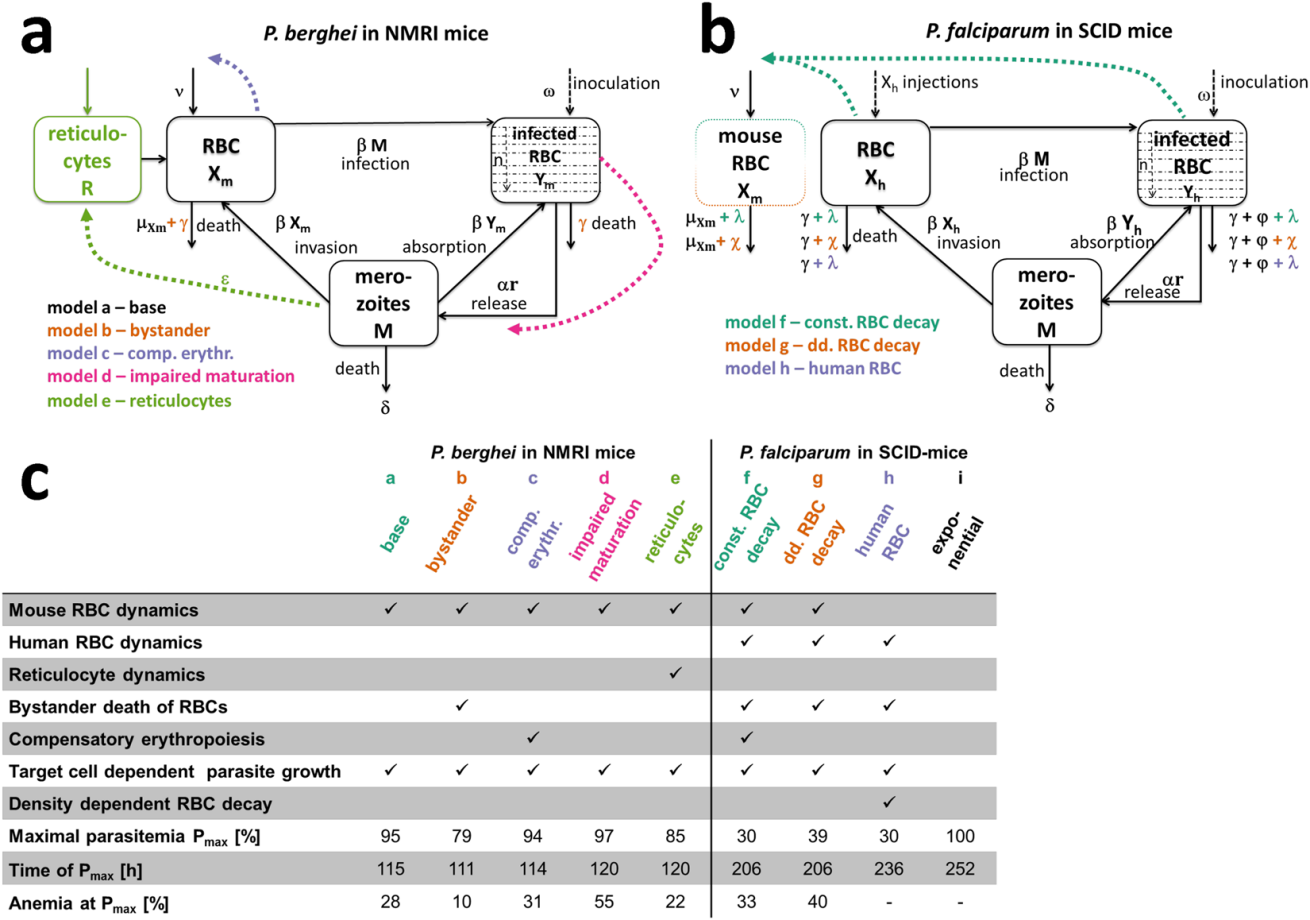
Schematic representations of the mechanistic within-host parasite growth models for *P. berghei* (**a**) and *P. falciparum* (**b**), with summary model details (**c**). The base model by^16^ is represented in black with model modifications added in color, for all models erythrocytic parasite stage was split into *n* age compartments (*n* = 12). *Model a* to *e* for *P. berghei* mainly capture processes dictated by the host-parasite system such as reactions of the host to increasing infection in *model b (bystander)*) and *model c (comp. erythr.)*, changes in parasite dynamics over the course of infection *model d (impaired maturation*), and host cell preferences of the parasite *model e (reticulocyte)*. In turn, *model f* to *h* for *P. falciparum* dynamics are primarily influenced by the experimental set-up of continued human RBC injections. Whereas *model f (const. RBC decay)* and *g (dd. RBC decay)* additionally explicitly model mouse RBCs, *model h (human RBC)* only captures human RBC populations. RBC or parasite transitions are represented with solid lines and influencing processes with colored dashed lines. (c) Selected index numbers characterizing the growth of parasites in the respective mechanistic mouse models for the experiment shown in Fig. 3. Anemia is defined as the percentage of RBCs compared to values prior to infection.

We developed five mathematical models of parasite growth for *P. berghei* (*models a* to *e*) and four models for *P. falciparum* in SCID mice (*models f* to *i*). Each model captures different levels of details and assumptions concerning RBC dynamics of the host (NMRI or SCID mice), the influence of the parasite on RBC dynamics, and parasite growth characteristics (Fig. 1).

A previous within-host model capturing RBC and parasite dynamics^16^ described by a set of ordinary differential equations (ODEs) was used as our base model (*model a*) for our *P. berghei* and *P. falciparum* growth models. This model captures parasite growth as well as RBC dynamics. It assumes constant production υ [cells/h] and decay μ [1/h] of healthy RBCs X^16^, that are infected by merozoites M dependent on the infectivity parameter β [cells/mL h]. Infected RBCs Y burst on average after one parasite life cycle 1/α h later and subsequently release r new merozoites that die with rate δ [1/h]. In contrast to^16^, for all *models a* to *h*, the intra-erythrocytic parasite stage was split into *n* age compartments (*n* = 12, based on stability analysis of the base model structure (Supplementary Fig. S5)) with a transition rate of α_n_ = α n [1/h] between compartments. Although *models b* to *h* can be considered as expansion of *model a (base)*, we deliberately illustrate them separately to compare model conclusions regarding influences of parasite-host dynamics on drug efficacy estimates. Therefore, we decided against nested model building.

In addition to our base model, we accounted for other parasite host interactions for *P. berghei*, whereby *model b (bystander)* included a bystander-death rate γ [1/h] of uninfected RBCs caused by the reaction of the innate immune system to parasite growth^31^. Compensatory erythropoiesis, caused by anemia through RBC destruction, was considered in *model c (comp. erythr.)*^31,32^. Potential changes to parasite properties was examined in *model d (impaired maturation)* assuming an increase in parasite densities causes lengthening of the intra-erythrocytic parasite life cycle from 24 to 37 h^33^. *Model e (reticulocytes)* allowed for an age preference of the parasite by including immature RBC (reticulocyte) dynamics^31^.

In order to adequately represent RBC and parasite dynamics as a consequence of the continued RBC injections, we extended and adapted *model a (base)* for *P. falciparum* growth in SCID mice by including human RBC dynamics in *models f* to *h*. To capture base decay rates of mouse and human RBCs, we assumed a constant decay rate λ [1/h] in *model f (const. RBC decay)* and model *h (human RBC)* as well as total RBC density-dependent (dd.) decay χ [1/h] in *model g (dd. RBC decay*) as a mouse reaction to continued RBC injections^11^. Additionally, we implemented parasite density-dependent clearance of RBCs by phagocytes γ [1/h] to account for infection-induced dynamics of RBC clearance and splenic/liver clearance φ [1/h] in all *models f* to *h*. While *model f* and *g* included human and mouse RBC dynamics, *model h (human RBC)* assumed mouse RBC dynamics to be negligible and only captured human RBC dynamics. Our last model, empirical *model i (exponential)*, assumed exponential parasite growth without explicit host-parasite dynamics and no resource depletion.

For all mechanistic *models a* to *h*, we assumed that parasite age was uniformly distributed at time of inoculation and asynchronous parasite growth based on previous descriptions of *P. berghei ANKA* infections desynchronizing after inoculation^34^. Data resolution was too low to inform models of synchronous parasite growth in SCID mice. Since the *P. berghei ANKA* strain in NMRI mice is very aggressive (fatal within six days) and there is no fully functioning immune system in SCID mice, the dynamics of the adaptive immune system were not considered in either mice system.

The mechanistic *models a* to *h* used the time of parasite inoculation as a starting point for modeling whereas time of drug administration (72 h post-infection) was chosen as the start of the exponential growth phase for *model i (exponential)* due to data availability. Data was pooled per experiment for all parameter estimations with an experiment defined as a group of mice having the same control group. No individual parameter estimation per mouse was conducted. An overview of our modeling workflow, model ODEs and specifications of parameters estimated or fixed to literature and experimental values are given in the Supplement.

### Model fits to data

*Models a* to *h* were able to account for changes in experimental setting by using experimental information on parasite inoculation time and amount, and RBC injections, as input to the models. Additionally, for *model f* and *g*, the initial percentage of human RBCs H_0_ was estimated per experiment, while constrained to value ranges extracted from laboratory protocols.

**Figure 2:**
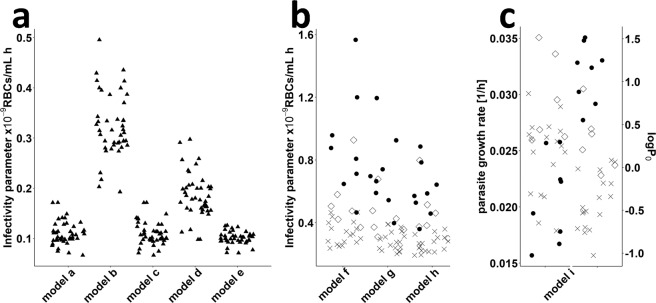
Estimated values of the infectivity parameter β by model for both murine experimental systems. Each symbol represents the value estimated for one experiment. (**a**) Values estimated for murine *P. berghei* infection. *Model a (base)*, *c (comp. erythr.)* and *e (reticulocyte)* show similar results whereas higher values were estimated for *model b (bystander)* and *d (impaired maturation)* (**b**) Estimated β –values for infection of humanized mice with *P. falciparum using the mechanistic models f (const. RBC decay)*, *g(dd. RBC decay)*, and *h (human RBC)*. (**c**) Parasite growth rate p_gr_ and parasitemia at start of the exponential growth phase P_0_ (72 h post-infection) estimated for *model i (exponential)*. The laboratories are denoted by different symbols (not identified here).

The differences in experiments are likely a consequence of variation in laboratory procedures, such as thawing of parasites, age and infection status of the donor mouse, altered parasite virulence due to serial passage of the parasite, or inoculum size. We therefore assessed the ability of either the infectivity parameter β (which effectively represents differences in parasite fitness and virulence) or the viability of the parasite inoculum ω (representing differences in thawing protocols) to capture differences between experiments. The infectivity parameter β was able to account for observed differences in force of parasite growth between experiments and laboratories. By comparing β-values, we found consistent differences between each laboratory and model (Fig. 2). Our estimates of β for *P. berghei* and *P. falciparum* range from 6.7 × 10^−11^ to 5.0 × 10^−10^ and 1.2 × 10^−10^ to 1.6 × 10^−9^ cells/mL/h, respectively. For *model i (exponential)* an adjustment of base parasitemia P_0_ at treatment start and parasite growth rate p_gr_ was necessary to capture inter-experimental differences. Estimates of parasite growth rates for *model i* range between 0.016 and 0.035 [1/h] (Fig. 2).

Even though the mechanistic parasite growth *models a* to *e* for *P. berghei* and *models f* to *h* for *P. falciparum* showed similar parasite growth patterns in their respective murine hosts, several distinct characteristics affecting parasite growth and treatment patterns became apparent in our analysis.

**Figure 3:**
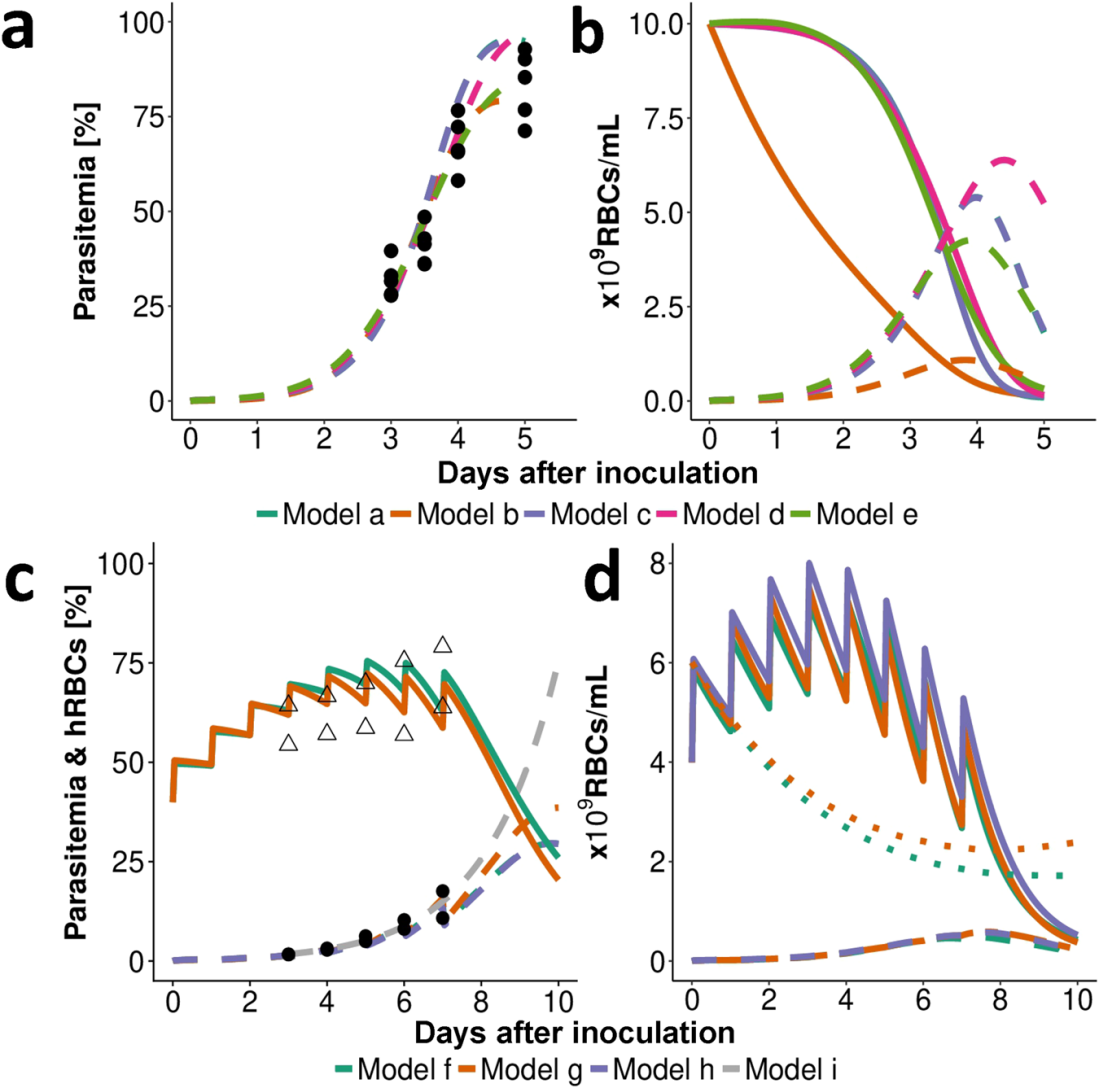
Representative fit of the within-host models to data. (**a**) Data (•) and model predictions (- -) of infection with *P. berghei* with an inoculum of 2 × 10^7^ infected RBCs (i.v.) show a steep increase in parasitemia three days after inoculation. (**b**) Model output for unobserved total numbers of RBCs show an increase in infected RBCs (- -) with a simultaneous decrease in uninfected RBCs (▬) resulting in anemia. However, the total number of human and murine RBC populations differs between model predictions (compare *model b (bystander)*), given that the estimated percentage of infected RBCs is compared to observed. Further differences in models become apparent comparing predicted time of, and total parasite numbers at, peak parasitemia P_max_ (see Fig. 1c). (**c**) Infection of SCID mice with *P. falciparum* through an inoculum of 3.5 × 10^7^ infected RBCs (i.v.). Human RBCs (∆) are injected daily until day seven post-infection increasing total human RBC counts (▬). (**d**) As uninfected RBCs (▬) increase the predicted number of mouse RBCs (• •) decrease due to random clearance of excess RBCs. After RBC injections are ceased, the model predicts a steep decline in human RBCs. Data (•) and *models f* to *h* (- -) show lower values of predicted peak parasitemia compared to *model i*.

As expected, a steep decline of uninfected RBCs was predicted with increasing parasite load of *P. berghei* (Fig. 3). This resulted in anemia, defined as the percentage of RBCs compared to values prior to infection, of up to 10% (Fig. 1). Dependent on model choice, different time courses for total (un)infected parasite populations were observed (Figs. 1c and 3b) resulting in a range of maximal parasitemia values between 79% and 97%. Continued human RBC injections prevent the occurrence of anemia during *P. falciparum* infection. However, if human RBC injections cease *models f* to *h* predicted a steep decrease in human RBCs, also observed in laboratory experiments (Supplementary Fig. S4), emphasizing the importance of capturing experimental RBC replenishment and clearance mechanisms. Compared with base clearance of mouse RBCs (0.001 [1/h], Supplementary Table S4) we estimated base clearance of human RBCs to be increased by a factor of 10 with *model f (const. RBC decay)* and *model h (human RBC)* estimating λ of 0.01 and 0.08 [1/h] and *Model g (dd. RBC decay)* estimating a maximum base clearance χ_max_ of 0.018 and kχ_,50_ of 1.05 × 10^10^ RBCs/mL (Supplementary Table S7). Total RBC counts tracked by *model f (const. RBC decay)* and *model g (dd. RBC decay)* reached a maximum value of 1.2 × 10^10^ RBCs/mL (*model h*; mouse RBCs not considered). The base death rate of RBCs λ was estimated to be smaller than the maximum parasite density-dependent death rate γ_max_ for all models.

### Drug action models and predicted translation between murine systems

**Figure 4:**
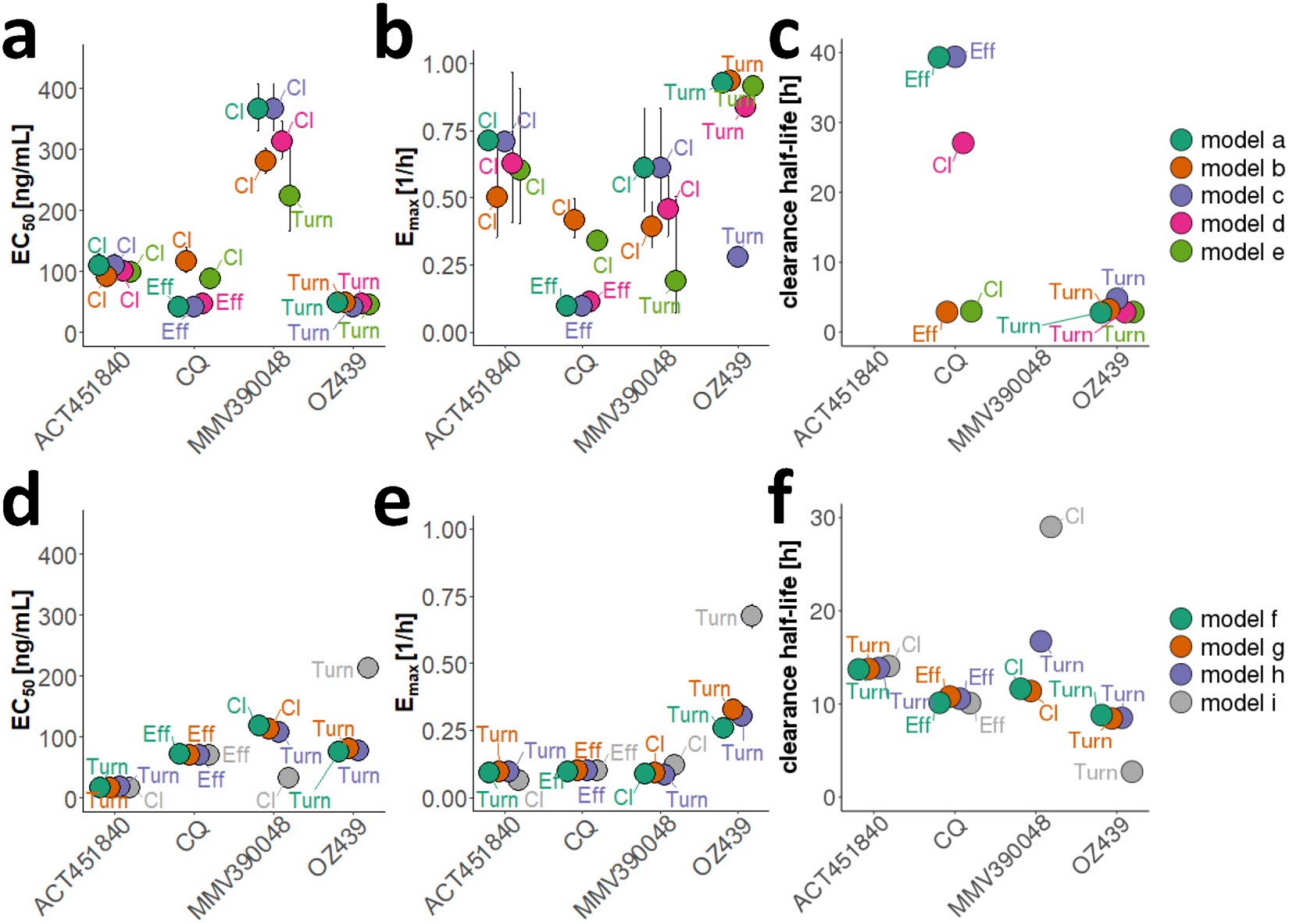
Comparison of drug efficacy estimates found for *P. berghei* in normal mice (**a–c**) and *P. falciparum* in SCID mice (**d–f**). EC_50_ [ng/mL], E_max_ [1/h] and the clearance half-life [h] are illustrated for each drug and parasite growth model. The drug action model showing the best fit to data was chosen based on ΔOFV (AIC), visual assessment of model fit and biological plausibility for each parasite growth model (with Turnover-model (Turn), drug action through an effect compartment (Eff) and delayed clearance of dead parasites (Cl)). See Supplementary Table S9 for parameter values.

Following our workflow detailed in Fig. S1, the parasite growth models were combined with compartmental PK models to investigate drug efficacy. The change in parasite death rate α was chosen as the pharmacological action for PD models (Supplementary Table S6). Parameters of parasite growth and PK models were fixed to previously estimated values (Supplementary Tables S7 and S8) for calibration of EC_50_, E_max_ and additional parameters describing drug action models (Supplementary Table S6) against treatment data. We compared EC_50_, E_max,_ and the structural PD models across murine systems and parasite growth models to assess influences of parasite-, host- and drug-interactions on drug efficacy analysis (Fig. 4, parameter values in Supplementary Table S9) and to investigate potential translation between murine systems.

**Figure 5:**
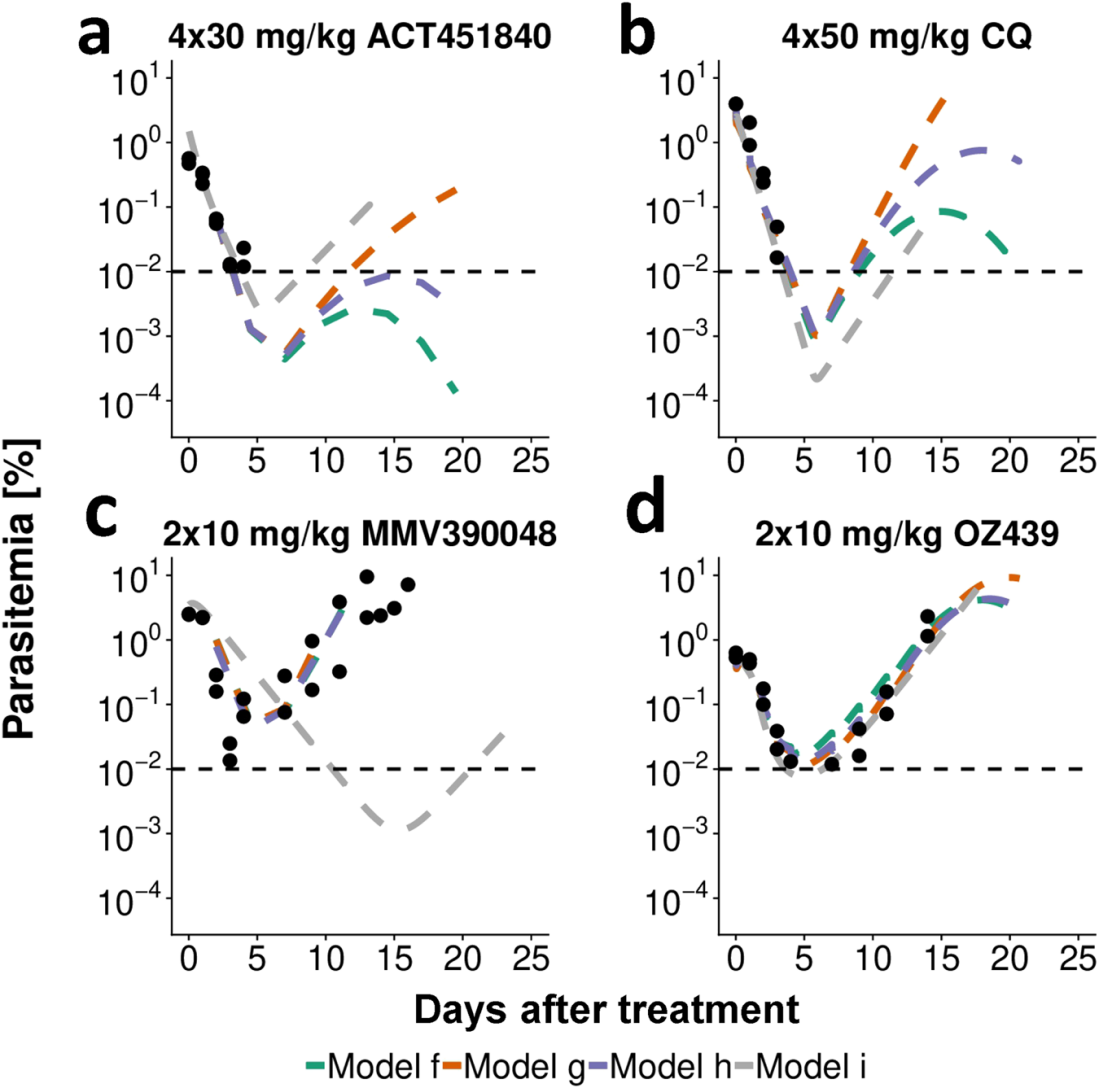
Representative fits of drug action models in SCID mice infected with *P. falciparum* at day 0 with an inoculum of 2 × 10^7^–3.5 × 10^7^ infected RBCs. The models were fitted to data of all administered doses with model predictions for the respective doses portrayed here. Treatment commenced three days after inoculation in dosing intervals of 24 hours. Mice were treated with 4 × 30 mg/kg ACT-451840, 4 × 50 mg/kg CQ, 2 × 10 mg/kg MMV390048 or 2 × 10 mg/kg OZ439. The cessation of human RBC injections in ACT-451840 and CQ experiments seven days after treatment leads to a decay of human RBCs and therefore also parasitemia 10–15 days after treatment (**a,b**). The horizontal dashed line represents the lower limit of quantification with 0.01% parasitemia. n = 2 mice for all doses shown.

A typical fit of parasite growth and drug action models to treatment data of SCID mice is shown in Fig. 5. As expected, all models predicted decreasing parasite counts following non-curative treatment until an inflection point after which parasite counts increased again. This inflection point is generally below the lower limit of quantification (LLOQ) (microscopic detection limit: 0.01% parasitemia) for effective treatment (Fig. 5). Structural drug action models were compared for each combination of parasite growth model, murine system and drug (lowest AIC, Supplementary Fig. S2). In *P. berghei* infection, at least three out of five and in *P. falciparum* infection at least three out of four parasite growth models match with respect to the chosen structural PD model (Fig. 4). All PD models either implement a delayed drug effect through an effect compartment or turnover model, or delayed clearance of dead parasites.

Although all drugs were active in both mouse systems, no apparent linear relationship was found when comparing estimated EC_50_, E_max_, and parasite clearance half-life values of the four drugs investigated between both murine experimental systems given chosen model of drug action and associated parameters (Supplementary Table S9). Parasite clearance half-life was generally lower for treatment of *P. berghei*.

### Recrudescence

**Figure 6:**
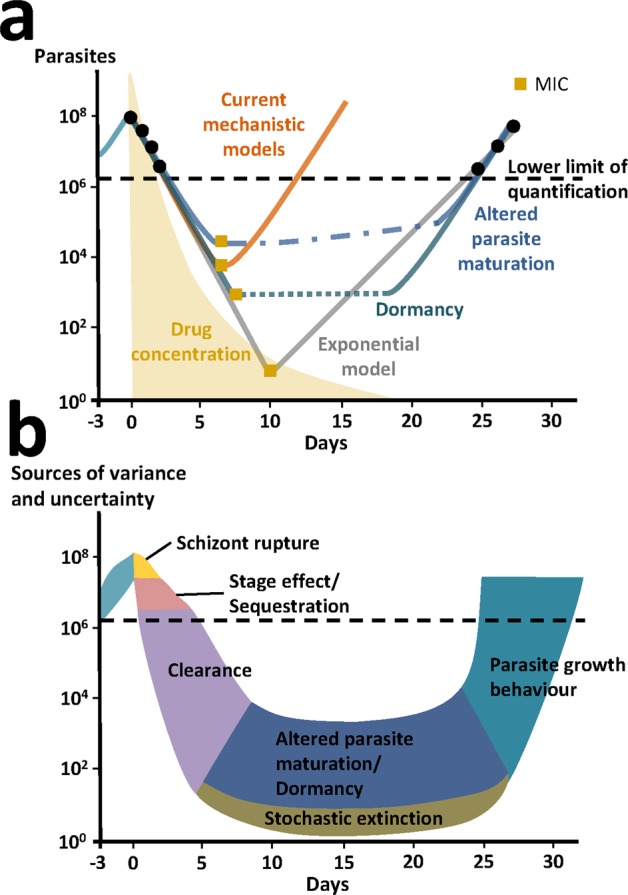
Schematics of *P. falciparum* parasite dynamics in SCID mice after treatment and potential factors explaining variance and uncertainty. (**a**) The mechanistic models (orange) presented in this paper assume parasite growth characteristics remain constant throughout treatment and are therefore not capturing late recrudescence. This is in contrast to the exponential model (gray) that compensates for late recrudescence by shifting the curve to low parasite and drug concentrations. Alternative to our mechanistic models, we propose some hypothetical parasite recrudescence curves (blue and green), that include additional phenomena such as altered parasite maturation and parasite dormancy offering possible explanations for late recrudescence. We cannot capture these mechanisms with models without additional data. MIC estimates important for experimental interpretation and translation to humans are shown by yellow square points and are likely to be very different given assumptions about parasite growth behavior after treatment. (**b**) We hypothesize and extended the sources of variance and uncertainty of the parasite treatment curve described in^35^ to schematically illustrate parasite phenomena during growth, treatment and recrudescence for antimalarial experiments (murine and possibly human). These extended phenomena include altered parasite maturation, dormancy, and stochastic extinction occurring below the lower limit of quantification hindering estimation.

For the long-lasting recrudescence experiments conducted with MMV390048 and OZ439 in SCID mice, we were only able to capture recrudescence (occurring more than eight days after last measured parasitemia above the lower limit of detection) with the exponential growth *model i*. Although we capture recrudescence within eight days of last measurement, we were unable to describe recrudescence after this time, with the mechanistic parasite growth *models f* to* h* (Supplementary Fig. S3). Minimum parasite numbers predicted by the mechanistic growth models remain high, e.g., after dosing with 1 × 50 mg/kg OZ439, total parasite numbers of 3.3 × 10^6^ parasites (≈0.02% parasitemia) are predicted by *model f (const. RBC decay)*. In contrast, *model i (exponential)* predicts a minimum parasitemia of 0.0003%. This indicates that additional parasite phenomena such as altered parasite maturation, dormancy, or stochastic extinction might be at play that have not been considered in the mechanistic models (Fig. 6: extension sources of variance and uncertainty of the parasite treatment curve described in^35^ applicable to murine and human infections for antimalarial investigations). The inclusion of these additional parasite characteristics is also potentially influencing minimum inhibitory concentration (MIC) definition and estimates (Fig. 6a).

## Discussion

By simultaneously capturing parasite growth and treatment, our mechanistic models provided insight into the influence of interactions between host, parasite, drug, and experimental background in preclinical murine systems for assessing existing and novel antimalarials. Mechanistic modeling and simulation enabled exploration of these host-parasite interactions along the preclinical development pathway to understand their potential effect on compound selection in preclinical models.

In general, we found that host-parasite dynamics and experimental set-up (e.g. in terms human RBC injections) had an influence on estimated parasite growth measured as parasite invasion rates, clearance and maturation rates, and the availability and replenishment of resources. Explicit inclusion of these mechanisms in our parasite growth models and subsequent analysis of translation of PD parameters and recrudescence identified the importance of considering dynamics of the murine system during analysis. The importance of host-parasite interactions for drug effect imply that careful consideration is needed to define and use appropriate mechanistic parasite growth models for translating, not only between murine systems, but also to humans and to predict human-equivalent dose.

We decided against nested parasite growth model building but rather we separately portrayed model predictions and fits and thus compared conclusions drawn in a non-weighted ensemble approach, acknowledging the different assumptions in each model. Model averaging was forgone to illustrate uncertainty concerning underlying parasite-host interactions over time and their influence on drug efficacy estimates. This was also important in order to highlight if further mechanistic insights are needed due to limited data per mouse and experiment.

To parameterize our models to all the available experimental data we needed to consider inter-experimental differences in the infectivity parameter β. Our estimates of β for *P. berghei* and *P. falciparum* are similar to those described in literature^9,18^. The variations in β, which effectively represent differences in parasite fitness and virulence, are likely a consequence of differences in laboratory procedures, such as thawing of parasites, age and infection status of the donor mouse, altered parasite virulence due to serial passage of the parasites and inoculum sizes.

We found properties of the host-parasite system to be the primary influence on undisturbed parasite growth of *P. berghei* in NMRI mice. Resource availability, in the form of RBCs, drives untreated parasite growth with mice exhibiting up to 90% peak parasitemia and 10% anemia five days post-infection. Similar anemia values ranging between 10 and 30% have been previously published^31,36^. As a consequence of RBC availability, *models a* to *e* predicted a decrease in total parasite densities after reaching peak parasite concentrations (Fig. 3b). Our analysis estimated that the preference of *P. berghei* for reticulocytes was less pronounced than found previously^31^. This discrepancy could be due to the previous study using a mouse strain that tolerated longer lasting infections, during which activation of erythropoiesis led to increased reticulocyte numbers. This increase in infection length could facilitate age preference of parasites to be measurable. The influence of parasite-host interaction, in form of impaired parasite maturation, was found to be most prominent in advanced infections resulting in later and higher peak parasitemia. These time dependent parasite characteristics should therefore be considered in experimental design considerations.

In contrast, in the SCID mouse system, parasite growth is primarily influenced by the artificial replacement of erythropoiesis with injections of mature human RBCs containing negligible numbers of reticulocytes. Therefore, the analysis of erythropoietic processes and age preferences of the parasite are rendered irrelevant. The impairment of parasite maturation has previously been attributed to host immune mechanisms regulating parasite growth during early stages of infection^33^. For this reason, changes in parasite maturation were likely not observed in immunodeficient rag1^−/−^mice^33^ and were therefore not considered in this analysis. To date the occurrence of this process in human and thus potential clinical implications remains unclear. To gain further insights into parasite-host dynamics influencing existing experiments, we suggest collecting additional data per experiment on total (un)infected RBC concentrations, and in SCID experiments on total mouse and human RBC concentrations. Resolution of present parasite age-stages could provide insights into parasite maturation dynamics. Additional *in vitro* experiments investigating recrudescence patterns after treatment with different drugs could inform the analysis of potential parasite dynamics below the LLOQ^37^. Influences of immunity on the efficacy of different drugs and recrudescence patterns could be assessed by utilizing chronic infection of mice^38^. The differences elucidated between parasite growth patterns in the respective systems of murine malaria infection were also reflected in the analysis of antimalarial action. A comparison of drug efficacy parameters between host-parasite systems did not allow a direct translation between systems. The variability of the drug action parameters EC_50_ and E_max_ between murine systems could be caused by previously discussed differences in host-parasite interactions such as erythropoiesis, the ability to cause chronic infections, the development of anemia and differing parasite characteristics. Additionally, differences between parasites species on a molecular level are likely influencing anti-parasitic activities of compounds that are specific inhibitors of enzyme activity such as ACT-451840 and MMV390048 (Supplementary Table S1). We conclude that while the absence of erythropoiesis, anemia, and a functioning immune system described in SCID mice allows for an unperturbed investigation of the sole drug effect, direct translatability of drug action parameters to humans could be complicated should these processes be of importance in human infection.

However, in SCID mice underlying clearance of (un)infected RBCs could influence the analysis of drug efficacy data. Our mechanistic SCID models break down overall decrease in parasitemia into clearance mechanisms induced by experimental set-up, murine experimental system, and drug action. We estimated similar value ranges in terms of clearance attributable to host-reactions to infection γ_max_ (0.055–0.44) (Supplementary Table S7) and drug action E_max_ (0.065–0.33) (Supplementary Table S9) across all drugs and drug action models. Commonly used measures of drug efficacy such as parasite clearance half-life^39^, summarize all parasite clearance in a single index number^40,41^ when in fact parasite clearance is the net effect of multiple parasite clearance mechanisms^23^. Therefore, the inclusion of delayed removal of parasites affected by the drug into mechanistic parasite growth models could prevent potential misinterpretation of parasite clearance half-life estimates. Analysis of more drugs and routine measurement of (stage-specific) parasite clearance rates would give valuable insight into clearance mechanisms and prevent misinterpretation of parasite clearance after treatment.

We observed interactions between parasite and host system that resulted in parasite growth characteristics changing over time and with subsequent influence on observed drug efficacy. Thus, it is important to discuss the mechanisms of parasite recrudescence below the lower limit of quantification. Simple linear regression analysis showed a statistically significant prediction (p = 0.0442 after MMV390048 and p = 6.8e-5 after OZ439 treatment) of recrudescence times using the slope of the parasite treatment curve, number of drug doses, and dose (Supplementary Tables S11 and S12). Our results indicate positive correlation between high drug exposure due to increasing doses and regimens and the time to recrudescence. The low proportion of explained variance (R^2^) for MMV390048 may be caused by the data capturing both, alive and dead parasites due to the clearance model best describing drug action of MMV390048 in SCID mice (Supplementary Table S9). However, the mechanistic parasite growth models in SCID mice were not able to capture the range of incidence and times of recrudescence observed between and within experiments (Supplementary Table S10 and Fig. S3). Although parasite recrudescence generally occurred at later times with increasing doses in our models, time of recrudescence could not be mechanistically explained. Apart from variability in drug efficacy parameters such as EC_50_ and E_max_, additional pharmacological or parasitic processes such as parasite dormancy^42,43^, impaired parasite maturation^44,45^, altered parasite clearance^44^, and additional stochastic effects may be delaying recrudescence (Fig. 6). Previous studies indicated links between parasite virulence^38,46^, parasite numbers at time of treatment^38,46^, and treatment duration^38,46^ as influencing frequency of recrudescence for different *Plasmodium* parasites. Overall, these findings suggest, that the current mechanistic models do not provide additional structural insight to late recrudescence (Fig. 6).

In comparison to mechanistic growth models, the exponential growth *model i* was not helpful in providing insights into mechanistic parasitic behavior and drug action. Parasite growth parameter estimates for *model i (exponential)* are based on the exponential growth phase three days after inoculation (as no data is collected before), whereas the mechanistic models start at time of inoculation (using inoculum size) and therefore account for potential growth lag phases. Consequentially, the exponential model predicts a biologically implausible instant switch from drug suppressed growth to exponential growth after non-curative treatment. We found the direct influence of drug action on the estimated parasite growth parameter p_gr_, which combines parasite growth and death, facilitates shifts of predicted recrudescence curves to fit recrudescence data (Fig. 6a). These lacks of mechanistic insights in the exponential model warrant caution in drawing conclusions from drug efficacy indices (e.g. MIC) derived directly from this model to translate to human clinical phases^9^. In contrast, hypothetical growth curves including parasite dormancy and altered parasite maturation depicted in Fig. 6 allude to the fact that changes in growth behavior are not captured in current models and that the MIC might not be a single concentration but rather a concentration range (dotted lines). Investigations into clinical relevance of recrudescence mechanisms in humans might be worthwhile to forecast treatment efficacy in the field^47–49^. To date, neither mechanistic parasite growth models nor exponential growth models have been validated for human dose prediction. Further understanding of mechanistic background is necessary to understand their respective suitability and appropriate use cases for model simplification.

Despite our insights, our study comes with several limitations. Data availability and richness varied greatly between murine systems, experiments and antimalarials (Supplementary Table S2). Data per experiment was pooled as parasite density and drug concentration measurements and thus also parameter estimation could not be carried out per individual mouse due to constraints of the parasite-host system and experimental set-up (e.g. sampling frequency). Although we chose our model assumptions carefully based on current literature they are still simplifications of a complex system and do not fully capture the complexity of murine malaria infection, (e.g. synchronized growth of *P. falciparum* and antimalarial stage specificity).

To date, translation of drug efficacy parameters between experimental murine systems and humans is undertaken using PD parameters/indices such as parasite clearance estimator (PCE) or MIC. However, we demonstrated the influence of different mechanistic backgrounds of mouse systems and parasite clearance on drug efficacy estimates. Our analysis of parasite recrudescence behavior in the models compared to observed data indicates additional unknown mechanisms influence parasite recrudescence timing and thus highlights potential pitfalls in using MIC for human-equivalent dose prediction. Further research on the importance of these mechanistic insights in humans and translation of PD indices between preclinical and clinical phases using historical preclinical and clinical data of existing antimalarials could accelerate the drug development process. Given the current standard of translation and dosing recommendation we conclude that, for now, further analysis of modeling results from both, preclinical experimental systems offers great potential to support optimal treatment of humans.

## Materials and Methods

### Data

*In vivo* efficacy studies of *P. berghei ANKA*-infection were conducted at Swiss TPH as previously described^25,30^. Briefly, NMRI mice were infected with 2 × 10^7^ parasitized RBCs (i.v.), and treatment consisted of one to four doses (p.o.) commencing 4–72 h after infection. Parasitemia was measured 72 or 96 h after infection. Study outcomes are reduction in parasites compared to a control group, mouse survival, and mouse cure. In untreated mice, death usually occurs 6 d after infection.

*In vivo* efficacy experiments against *P. falciparum* Pf3D7^0087/N9^ in NOD ^scidIL-2R′ c−/–^mice were conducted at GSK (GlaxoSmithKline), Swiss TPH and TAD (The Art of Discovery). Mice were engrafted with human RBCs by continued injections of human blood suspension, and infected with *P. falciparum* after a hematocrit of 40–75% was established. Treatment commenced 72 h post-infection. Original experimental outcomes were reduction in parasitemia compared to a control group, mouse cure, and parasite-recrudescence behavior. Human blood injections were repeated every one to three days to maintain sufficient red blood cell levels and prevent occurrence of anemia throughout each SCID mouse experiment. For both murine systems, cure was defined as having no detectable parasites 30 days post-infection^10,11^. The microscopic limit of detection is 0.01%, as a direct consequence of the total number of erythrocytes monitored for infection (10,000).

In *P. berghei* experiments, a wide range of doses were commonly tested with one measurement point per mouse (72 or 96 h after inoculation). In contrast, fewer doses were tested with fewer mice per dose in *P. falciparum* experiments. However, parasitemia and hematocrit were measured multiple times (at least once a day on day three up to day seven post-infection), with experiments lasting up to 32 days. All provided data was compiled into a database containing experimental data, along with information concerning experimental set-up and laboratory.

The animal experiments performed were approved by the Swiss Cantonal Authorities or by the Diseases of the Developing World Ethical Committee on Animal Research. The animal studies carried out at GSK were in accordance with European Directive 2010/63/EU and the GSK Policy on the Care, Welfare and Treatment of Animals and were accredited by the Association for Assessment and Accreditation of Animal Laboratory Care for the ones performed at Diseases of the Developing World Laboratory Animal Science facilities. The animal experiments carried out at the Swiss Tropical and Public Health Institute (Basel, Switzerland) are adhering to local and national regulations of laboratory animal welfare in Switzerland. Protocols are regularly reviewed and revised following approval by the local authority (Veterinäramt Basel Stadt).

### Modeling workflow

We developed a modeling workflow applicable for systematic analysis of antimalarial drugs which spans data handling, model development, parameterization, and simulation. The workflow is illustrated in Supplementary Fig. S1. In brief, we firstly developed multiple within-host parasite-growth models for both murine systems based on parasite characteristics described in the literature and via experimental background and settings (RBC clearance and replenishment in SCID mice). Models were described by ODEs (Supplementary Table S3) and were parameterized using available untreated parasite growth data with several parameters extracted from literature (Supplementary Table S4). Secondly, we tested, selected, and parameterized appropriate PK models to the concentration-time profile for each drug given several tested doses. Thirdly, the undisturbed parasite growth model and the PK model were combined, and their parameters fixed, to estimate parameters of drug action using drug treatment data.

All parasite growth and PD modeling, data manipulation, and plotting was performed in R (Version 3.5)^50^ using the package IQRtools (Version 0.9.99)^51^. Parasite growth- and PD-parameters were estimated via a maximum likelihood approach on trust region optimization. Multiple estimation starting points were utilized in order to guarantee identification of the global minimum.

Growth model evaluation was performed using visual comparison of data to model output, and assessment of biological plausibility of all parameters for all models. Several PD drug action models were fitted for each drug and murine experimental system to capture direct effect of drug concentration as well as delayed effects via effect compartment or indirect response models (Supplementary Table S2), and selected by ΔOVF (AIC) (Supplementary Fig. S2). Hill coefficients were fixed to values between 1 and 7. The parasite clearance half-life was estimated from model simulations using the methodology described in^39^.

Several different PK models with varying number of compartments, absorption and clearance behavior were tested to identify the PK model best describing each of the four drugs investigated (see Supplementary Table S5). PK models were fitted to the concentration-time profiles simultaneously using nonlinear mixed-effects modeling in Monolix 2016R1^52^. The PK profile of each drug and murine system was chosen by comparing model AIC. Final PK models and parameter values can be found in the Supplementary Table S8.

## Supplementary information

Supplementary Information.

## Data Availability

The datasets analysed during the current study are available from the corresponding author on request and with permission of Medicines for Malaria Venture and Idorsia Pharmaceuticals Ltd.
