# Supplementary material for: Ensemble modeling highlights importance of understanding parasite-host behavior in preclinical antimalarial drug development
Source: Sci Rep. 2020 Mar 10;10:4410. doi: 10.1038/s41598-020-61304-8 (PMC7064600; doi:10.1038/s41598-020-61304-8)
Supplement: Supplementary file 1 — Supplementary Information. [file 41598_2020_61304_MOESM1_ESM.pdf]

Ensemble modeling highlights importance of understanding parasite-host behavior in preclinical antimalarial drug development

Authors:
Lydia Burgert[1,2], Matthias Rottmann[1,2], Sergio Wittlin[1,2], Nathalie Gobeau[3], Andreas Krause[4], Jasper Dingemanse[4], Jörg J. Möhrle[1,2,3], Melissa A. Penny*[1,2]

[1] Swiss Tropical and Public Health Institute, Basel, Switzerland

[2] University of Basel, Basel, Switzerland

[3] Medicines for Malaria Venture, Geneva, Switzerland

[4] Idorsia Pharmaceuticals Ltd, Clinical Pharmacology, Allschwil, Switzerland

*Corresponding author: melissa.penny@unibas.ch

Supplement

Section 1: Data and Drugs

Overview of compounds and data used for analysis of parasite growth *P. berghei* and *P. falciparum* in their respective murine host and drug efficacy experiments.

Table S1: List of compounds including compound characteristics

Drug	Molecule class	Mode of action	Literature
**ACT-451840**	phenylalanine-based	Interaction with multidrug resistance protein-1 (PfMDR1), needs further investigation	[1,2]
**CQ**	4-aminoquinoline	Inhibits heme polymerization in food vacuole	[3]
**MMV390048**	2-aminopyridine	Inhibits phosphatidylinositol 4-kinase (PfPI(4)K)	[3,4]
**OZ439**	peroxide	Peroxidative damage and oxidative stress through reductive activation by heme, needs further investigation	[5,6]

Table S2: Overview of data used in fitting parasite growth and drug action parameters (parasite density data), and concentration-time profiles (concentration time data). Doses are given in mg/kg.

	Data	Concentration time data	Parasite density data
***P. berghei* in normal mice**	Undisturbed parasite growth	-	No. mice: 215<br>No. experiments: 43<br>Mice per experiment: 2-5<br>Data source: Swiss TPH
	ACT-451840	No. mice: 12<br>No. experiments: 3<br>Mice per dose: 4<br>Quadruple dose: 100<br>Data source: Idorsia	No. mice: 69<br>No. experiments: 4<br>Mice per dose: 3-5<br>Single dose: 10, 15, 20, 25, 30, 60, 80, 100, 300<br>Triple dose: 3, 10, 2x15, 30, 50, 2x30, 100, 300<br>Data source: Idorsia
	CQ	No. mice: 2<br>No. experiments: 1<br>Mice per dose: 2<br>Single dose: 10, 100, 300<br>Data source: Swiss TPH	No. mice: 72<br>No. experiments: 8<br>Mice per dose: 2-5<br>Single dose: 3, 10, 30, 100<br>Quadruple dose: 3, 10, 30, 100<br>Data source: Swiss TPH
	MMV390048	No. mice: 9<br>No. experiments: 3<br>Mice per dose: 3<br>Single dose: 1, 10<br>Data source: MMV	No. mice: 65<br>No. experiments: 3<br>Mice per dose: 3-6<br>Single dose: 0.5, 1, 2, 3, 10, 25, 30<br>Quadruple dose: 0.3, 0.5, 0.8, 1, 3<br>Data source: Swiss TPH
	OZ439	No. mice: 6<br>No. experiments: 1<br>Mice per dose: 3<br>Single dose: 30, 100<br>Data source: MMV	No. mice: 220<br>No. experiments: 13<br>Mice per dose: 5-10<br>Single dose: 0.1, 0.3, 1, 1.5, 2, 3, 5, 10, 15, 20, 25, 30, 50, 100<br>Triple dose: 1, 3, 10<br>Data source: Swiss TPH
***P. falciparim* in NOD $^{scidIL-2R' c-/-}$ mice**	Undisturbed parasite growth	-	No. mice: 132<br>No. experiments: 32<br>Mice per experiment: 2-8<br>Data source: GSK, Swiss TPH, TAD
	ACT 451840	No. mice: 3<br>No. experiments: 1<br>Mice per dose:3<br>Single dose: 3<br>Data source: Idorsia	No. mice: 12<br>No. experiments: 1<br>Mice per dose: 3<br>quadruple dose: 3, 10, 30, 100<br>Data source: Idorsia
	CQ	No. mice: 4<br>No. experiments: 1<br>Mice per dose: 4<br>Single dose: 50<br>Data source: Swiss TPH	No. mice: 14<br>No. experiments: 3<br>Mice per dose: 2-3<br>Single dose: 50 mg/kg<br>Quadruple dose: 2, 5, 10, 50<br>Data source: Swiss TPH
	MMV390048	No. mice: 48<br>No. experiments: 7<br>Mice per dose: 1-2<br>Double dose: 1,10<br>Quadruple dose: 0.25, 0.5, 1, 5, 10, 20<br>Data source: GSK, TAD	No. mice: 50<br>No. experiments: 7<br>Mice per dose: 1-2<br>Double dose: 1,10,<br>Quadruple dose: 0.25, 0.5, 1, 5, 10, 20<br>Data source: GSK, TAD
	OZ439	No. mice: 34<br>No. experiments: 4<br>Mice per dose: 2<br>Single dose: 0.5, 1, 3, 5, 10, 20, 30, 50, 75, 100<br>Double dose: 25<br>Data source: GSK, Swiss TPH	No. mice: 48<br>No. experiments: 4<br>Mice per dose: 2<br>Single dose: 0.5, 1, 3, 5, 10, 20, 30, 50, 75, 100<br>Double dose: 25<br>Data source: GSK, Swiss TPH

Section 2: Mechanistic models

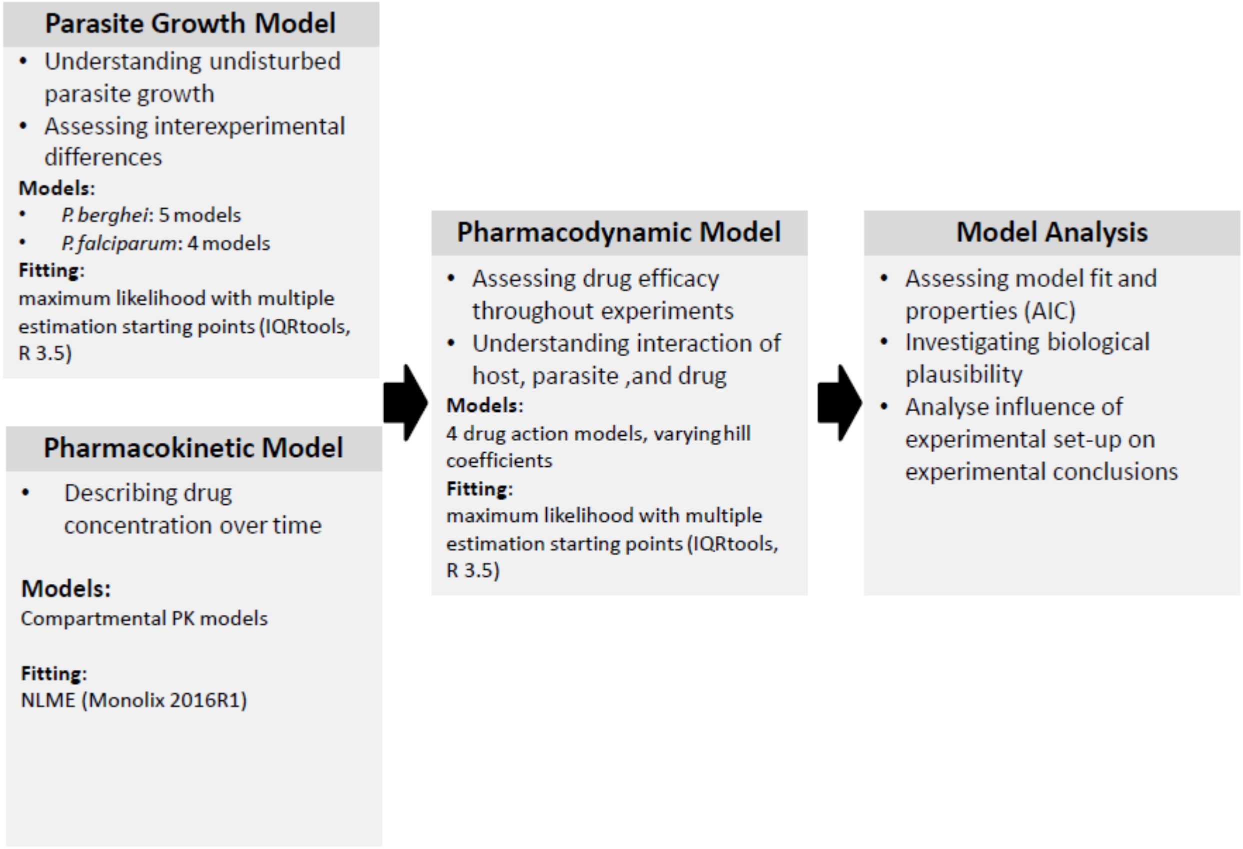

Figure S1: Standardized workflow for the systematic analysis of preclinical drug efficacy experiments. The workflow was designed to assist execution of consistent analysis of parasite growth and drug treatment experiments in a preclinical setting.

Table S3: Equations (ordinary differential equations) for the parasite growth models. Models are annotated in a modular construction system. The equation for uninfected (X) and infected (Y) RBCs and merozoites (M) for the respective model are listed in the table below. The annotations represent mouse RBCs (m), human RBCs (h) and reticulocytes (R). Parameter annotations can be found in Table S4.

Model a (base)	Model b (bystander)
$\frac{dX_m}{dt} = \nu - \mu_{X_m} X_m - \beta X_m M$ *(1)*<br>$\frac{dY_{Xm,1}}{dt} = \beta X_m M - \alpha Y_{Xm,1}$ (2)<br>$\frac{dY_{Xm,i}}{dt} = \alpha Y_{Xm,i-1}$- $\alpha Y_{Xm,i}$, $i = 2,..,n$ (2.1)<br>$\frac{dM}{dt} = -\beta(X_m + Y_{Xm})M + \alpha r Y_{Xm,n} - \delta M$ *(3)*<br><br>with: $Y_{Xm} = \sum_{i=1}^{n} Y_{Xm,i}$<br>$P = \frac{Y_{Xm}}{X_m + Y_{Xm}} 100$<br><br>and initial conditions: $X_{m,0} = X_{m,0}$, $M_0 = 0$<br>$Y_{Xm,0,i} = \omega \frac{\text{inoculum}}{Vn}$	$\frac{dX_m}{dt} = \nu - (\mu_{X_m} + \gamma)X - \beta X_m M$ (4)<br>$\frac{dY_{Xm,1}}{dt} = \beta X_m M - \alpha Y_{Xm,1}$ (2)<br>$\frac{dY_{Xm,i}}{dt} = \alpha Y_{Xm,i-1}$- $\alpha Y_{Xm,i}$, $i = 2,..,n$ (2.1)<br>$\frac{dM}{dt} = -\beta(X_m + Y_{Xm})M + \alpha r Y_{Xm,n} - \delta M$ (3)<br><br>with : $Y_{Xm} = \sum_{i=1}^{n} Y_{Xm,i}$<br>$\gamma = \frac{\gamma_{max} Y_{Xm}}{k\gamma_{50} + Y_{Xm}}$<br>$P = \frac{Y_{Xm}}{X_m + Y_{Xm}} 100$<br><br>and initial conditions: $X_{m,0} = X_{m,0}$, $M_0 = 0$<br>$Y_{Xm,0,i} = \omega \frac{\text{inoculum}}{Vn}$
**Model c (comp. erythr.)**	**Model d (impaired maturation)**
$\frac{dX_m}{dt} = \nu - \mu_{X_m} X_m - \beta X_m M$ (1)<br>$\frac{dY_{Xm,1}}{dt} = \beta X_m M - \alpha Y_{Xm,1}$ (2)<br>$\frac{dY_{Xm,i}}{dt} = \alpha Y_{Xm,i-1}$- $\alpha Y_{Xm,i}$, $i = 2,..,n$ (2.1)<br>$\frac{dM}{dt} = -\beta(X_m + Y_{Xm})M + \alpha r Y_{Xm,n} - \delta M$ (3)<br><br>with: $Y_{Xm} = \sum_{i=1}^{n} Y_{Xm,i}$<br>$\nu = \frac{\nu_{max}}{1 + \frac{X_m + Y_{Xm}}{k\nu_{50}}} X_{m,0}$<br>$P = \frac{Y_{Xm}}{X_m + Y_{Xm}} 100$<br><br>and initial conditions: $X_{m,0} = X_{m,0}$, $M_0 = 0$<br>$Y_{Xm,0,i} = \omega \frac{\text{inoculum}}{Vn}$	$\frac{dX_m}{dt} = \nu - \mu_{X_m} X_m - \beta X_m M$ (1)<br>$\frac{dY_{Xm,1}}{dt} = \beta X_m M - l Y_{Xm,1}$ (2)<br>$\frac{dY_{Xm,i}}{dt} = l Y_{Xm,i-1}$- $l Y_{Xm,i}$, $i = 2,..,n$ (2.1)<br>$\frac{dM}{dt} = -\beta(X_m + Y_{Xm})M + l r Y_{Xm,n} - \delta M$ (3)<br><br>with: $Y_{Xm} = \sum_{i=1}^{n} Y_{Xm,i}$<br>$l = \alpha - \frac{l_{max} Y_{xm}^2}{k_{l,50} X_{m,0} Y_{xm}^2}$<br>$P = \frac{Y_{Xm}}{X_m + Y_{Xm}} 100$<br><br>and initial conditions: $X_{m,0} = X_{m,0}$, $M_0 = 0$<br>$Y_{Xm,0,i} = \omega \frac{\text{inoculum}}{Vn}$

Table S3: Equations (ordinary differential equations) for the parasite growth models. *Continued…*

<u>Model e (reticulocytes)</u>

$$\frac{dR}{dt} = \nu - \tau_R R - \beta\varepsilon RM \quad (5)$$

$$\frac{dX_m}{dt} = \tau_R R - \mu_{X_m} X_m - \beta X_m M \quad (6)$$

$$\frac{dY_R}{dt} = \beta\varepsilon RM - \tau_{YR} Y_R - \alpha Y_R \quad (7)$$

$$\frac{dY_{Xm,1}}{dt} = \beta X_m M + \tau_{YR} Y_R - \alpha Y_{Xm}, 1 \quad (8)$$

$$\frac{dY_{Xm,i}}{dt} = \alpha Y_{Xm,i-1} \text{-} \alpha Y_{Xm,i}, \ i = 2, .., n \quad (8.1)$$

$$\frac{dM}{dt} = -\beta(X_m + Y_{Xm})M - \beta\varepsilon(R + Y_R)M + \alpha r Y_{Xm,n} - \delta M \quad (9)$$

with:

$$Y_{Xm} = \sum_{i=1}^{n} Y_{Xm,i}$$

$$P = \frac{Y_{Xm} + Y_R}{X_m + R + Y_{Xm} + Y_R} 100$$

and initial conditions:

$$R_0 = R_0$$

$$X_{m,0} = X_{m,0}$$

$$Y_{R,0} = 0$$

$$Y_{Xm,0,i} = \omega \frac{\text{inoculum}}{Vn}$$

$$M_0 = 0$$

<u>Model f (const. RBC decay)</u>

$$\frac{dX_h}{dt} = \text{inputRBC} - (\gamma + \lambda)X_h - \beta X_h M \quad (10)$$

$$\frac{dX_m}{dt} = \nu - (\lambda + \mu_{X_m})X_m \quad (11)$$

$$\frac{dY_{Xh,1}}{dt} = \beta X_h M - \alpha Y_{Xh,1} - (\gamma + \lambda + \varphi)Y_{Xh,1} \quad (12)$$

$$\frac{dY_{Xh,i}}{dt} = \alpha Y_{Xh,i-1} - (\gamma + \lambda + \varphi)Y_{Xh,i} - \alpha Y_{Xh,i}, \ i = 2, .., n \quad (12.1)$$

$$\frac{dM}{dt} = -\beta(X_h + Y_{Xh})M + \alpha r Y_{Xh,n} - \delta M \quad (13)$$

with:

$$Y_{Xh} = \sum_{i=1}^{n} Y_{Xh,i}$$

$$\nu = \frac{\nu_{max}}{1 + \frac{X_h + X_m}{k\nu_{50}}}$$

$$\gamma = \frac{\gamma_{max} Y_{Xh}}{k\gamma_{50} + Y_{Xh}}$$

$$P = \frac{Y_{Xh}}{X_h + X_m + Y_{Xh}} 100$$

$$H = \frac{X_h + Y_{Xh}}{X_h + X_m + Y_{Xh}} 100$$

and initial conditions:

$$X_h = H_0 10^{10}$$

$$X_{m,0} = (1 - H_0)10^{10}$$

$$Y_{Xh,0,1} = \omega \frac{\text{inoculum}}{Vn}$$

$$M_0 = 0$$

Table S3: Equations (ordinary differential equations) for the parasite growth models. *Continued…*

<u>Model g (dd. RBC decay)</u>

$$\frac{dX_h}{dt} = inputRBC - (\gamma + \lambda)X_h - \beta X_h M \quad (10)$$

$$\frac{dX_m}{dt} = \nu - (\lambda + \mu_{X_m})X_m \quad (11)$$

$$\frac{dY_{Xh,1}}{dt} = \beta X_h M - \alpha Y_{Xh,1} - (\gamma + \chi + \varphi)Y_{Xh,1} \quad (12)$$

$$\frac{dY_{Xh,i}}{dt} = \alpha Y_{Xh,i-1} - (\gamma + \chi + \varphi)Y_{Xh,i} - \alpha Y_{Xh,i}\ ,\ i = 2,..,n \quad (12.1)$$

$$\frac{dM}{dt} = -\beta(X_h + Y_{Xh})M + \alpha r Y_{Xh,n} - \delta M \quad (13)$$

with:

$$Y_{Xh} = \sum_{i=1}^{n} Y_{Xh,i}$$

$$\gamma = \frac{\gamma_{max} Y_{Xh}}{k\gamma_{50} + Y_{Xh}}$$

$$\chi = \frac{\chi_{max} N}{k\chi_{,50} + N},\ N = X_h + X_m + Y_{Xh}$$

$$P = \frac{Y_{Xh}}{X_h + X_m + Y_{Xh}} 100$$

$$H = \frac{X_h + Y_{Xh}}{X_h + X_m + Y_{Xh}} 100$$

and initial conditions:

$$X_h = H_0 10^{10}$$

$$X_{m,0} = (1 - H_0)10^{10}$$

$$Y_{Xh,0,i} = \omega \frac{inoculum}{Vn}$$

$$M_0 = 0$$

<u>Model h (human RBC)</u>

$$\frac{dX_h}{dt} = inputRBC - (\gamma + \lambda)X_h - \beta X_h M \quad (10)$$

$$\frac{dY_{Xh,1}}{dt} = \beta X_h M - \alpha Y_{Xh,1} - (\gamma + \lambda + \varphi)Y_{Xh,1} \quad (12)$$

$$\frac{dY_{Xh,i}}{dt} = \alpha Y_{Xh,i-1} - (\gamma + \lambda + \varphi)Y_{Xh,i} - \alpha Y_{Xh,i}\ ,\ i = 2,..,n \quad (12.1)$$

$$\frac{dM}{dt} = -\beta(X_h + Y_{Xh})M + \alpha r Y_{Xh,n} - \delta M \quad (14)$$

with:

$$Y_{Xh} = \sum_{i=1}^{n} Y_{Xh,i}$$

$$\gamma = \frac{\gamma_{max} Y_{Xh}}{k\gamma_{50} + Y_{Xh}}$$

$$P = \frac{Y_{Xh}}{X_h + Y_{Xh}} 100$$

and initial conditions:

$$X_h = H_0 10^{10}$$

$$Y_{Xh,0,i} = \omega \frac{inoculum}{Vn}$$

$$M_0 = 0$$

<u>Model i (exponential)</u>

$$\frac{dP}{dt} = p_{gr} P \quad (15)$$

with initial condition:

$$P_o = P_o$$

Table S4: Overview of model parameters of the mechanistic parasite growth models developed. Estimated parameters are denoted with Est. in the value column. (uninfected: uninf., infected: inf., calculated: calc., concentration: conc., erythrocyte: RBC)

**Process**	**Model**	**Para-meter**	**Value**	**Unit**	**Description**	**Ref**
Mouse RBC dyn.	a-c, f, g	ν	1.04×10^{7}	[cells/mLh]	Rate of RBCs production	[7]
Mouse RBC dyn.	a-g	μ_{χ_m}	1.04×10^{-3}	[1/h]	Death rate of uninf. RBCs	[7]
Mouse RBC dyn.	c, f	ν_{max}	2.00×10^{-3}	[1/h]	Maximum effect of total RBC conc. on ν	
Mouse RBC dyn.	c, f	$k\nu_{50}$	1e10	[cells/mL]	Concentration of total RBC concentration achieving 0.5 ν_{max}	
P. berghei dyn.	a-c, e	α	0.042 n	[1/h]	Death rate of infected RBCs in each age stage	[7]
P. berghei dyn.	a-h	δ	0.5	[1/h]	Death rate of merozoite	[7]
P. berghei dyn.	e	τ_R	0.014	[1/h]	Maturation rate of uninf. reticulocytes	[8]
P. berghei dyn.	e	ε	Est.	[]	Attraction of parasite to reticulocyte	[8]
P. berghei dyn.	e	τ_{YR}	0.042	[1/h]	Maturation rate of inf. reticulocytes	[8]
P. berghei dyn.	d	α_0<br>$k_{l,50}$<br>l_{max}	0.042 n<br>Est.<br>0.2	[1/h]<br>[cells/mL]<br>[1/h]	Base death rate of infected RBCs<br>Conc. of Y_{χ_m} achieving $0.5 l_{max}$<br>Maximum death rate of inf. RBCs	[7]
P. falc. dyn.	f, h	λ	Est.	[1/h]	Base death rate of all RBCs	
P. falc. dyn.	g	χ	Est.	[1/h]	Total RBC conc. induced clearance of RBCs	
P. falc. dyn.	g	χ_{max}	Est.	[1/h]	Maximum effect of total RBC concentration on χ	
P. falc. dyn.	g	$k\chi_{,50}$	Est.	[cells/mL]	Total RBC conc. achieving 0.5 χ_{max}	
P. falc. dyn.	i	p_{gr}	Est.	[1/h]	Replication rate of parasite in exponential growth phase	
P. falc. dyn.	f-h	α	0.025 n	[1/h]	Death rate of inf. RBCs at each age stage	[9]
Parasite dyn.	a-h	n	12	-	Number of modeled parasite age stages	
Parasite dyn.	a-h	ω	Est.	[]	Viability of parasite inoculum	
Parasite dyn.	a-h	β	Est.	[cells/mLh]	Rate of merozoites- RBC contact resulting in infection (infectivity)	
Parasite dyn.	a-h	r	Est.	[]	Number of merozoites per inf. RBC	
Parasite dyn.	b, f-h	γ	Est.	[1/h]	Infection induced RBC clearance	
Parasite dyn.	b, f-h	γ_{max}	Est.	[1/h]	Maximum effect of inf. RBCs on γ	
Parasite dyn.	b, f-h	$k\gamma_{50}$	Est.	[cells/mL]	Conc. of inf. RBCs resulting $0.5\gamma_{max}$	
Parasite dyn.	f-h	φ	Est.	[1/h]	Base clearance of inf. RBCs	

Table S5: Overview of model parameters of the mechanistic parasite growth models developed. . *Continued…*

Process	Model	Variable	Value	Unit	Description	Ref
RBC dyn.	a-g	X_m,<br>X_h	-	[cells/mL]<br>[cells/mL]	Mouse RBC conc.<br>Human RBC conc.	
Parasite dyn.	a-e	Y_{Xm},<br>Y_R,<br>Y_{Xh}.	-	[cells/mL]<br>[cells/mL]<br>[cells/mL]	Inf. mouse RBC conc.<br>Inf. reticulocyte conc.<br>Inf. human RBC conc.	
RBC. dyn	e	R		[cells/mL]	Reticulocyte conc.	
Parasite dyn.	a-h	M	-	[cells/mL]	Merozoite conc.	
Parasite dyn.	a-h	P	-	[%]	Parasitemia, Percentage inf. RBCs	
RBC dyn.	f-g	H	-	[%]	Hematocrit; Percentage human RBCs	
Mouse RBC dyn.	a-g	$X_{m,0}$	10^{10}<br>or Calc.	[cells/mL]	Initial number of mouse RBCs	
RBC dyn.	a-h	$X_{h,0}$	Calc.	[cells/mL]	Initial number of human RBCs	
Mouse RBC dyn.	e	R_0	5^8	[cells/mL]	Initial number of reticulocytes, 5% of $X_{m,0}$	
RBC dyn.	f-h	H_0	Est.	[%]	Initial percentage of human RBCs	8
Parasite dyn.	i	P_0	Est.	[%]	Initial parasitemia	
Model input	a-h	inoculum	Data	[c]	Parasite Inoculum	
Model input	f-h	inputRBC	Data	[c]	RBC injections	
Mouse RBC dyn.	a-h	V	1.23	[mL]	Mouse blood volume	10

Table S6: Structural pharmacokinetic models. Models were built in a nested manner. No covariate model was developed. Models were evaluated based on difference in AIC and standard VPCs. Such that the one compartment model corresponding to the absorption and elimination strategies in the Table S6, Q_1 and V_{p1} were set to zero.

Absorption	Elimination	Equation (2 compartments)
**1st order**	linear	$\frac{dAd}{dt} = -k_a A_d + Input$<br>$\frac{dA_c}{dt} = k_a A_d - \frac{Cl}{V_c} A_c - \frac{Q_1}{V_c} A_c + \frac{Q_1}{V_{p1}} A_{p1}$<br>$\frac{dA_{p1}}{dt} = + \frac{Q_1}{V_c} A_c - \frac{Q_1}{V_{p1}} A_{p1}$
**Michaelis-Menten**	linear	$\frac{dAd}{dt} = -\frac{v_{max} A_d}{V_{max,50+A_d}} + Input$<br>$\frac{dA_c}{dt} = \frac{v_{max} A_d}{V_{max,50+A_d}} - \frac{Cl}{V_c} A_c - \frac{Q_1}{V_c} A_c + \frac{Q_1}{V_{p1}} A_{p1}$<br>$\frac{dA_{p1}}{dt} = + \frac{Q_1}{V_c} A_c - \frac{Q_1}{V_{p1}} A_{p1}$
**linear**	Linear and Michaelis-Menten	$\frac{dAd}{dt} = -k_a A_d + Input$<br>$\frac{dA_c}{dt} = k_a A_d - \frac{Cl}{V_c} A_c - \frac{Q_1}{V_c} A_c + \frac{Q_1}{V_{p1}} A_{p1} - \frac{v_{max} A_d}{V_{max,50+A_d}}$<br>$\frac{dA_{p1}}{dt} = + \frac{Q_1}{V_c} A_c - \frac{Q_1}{V_{p1}} A_{p1}$

Parameter	Unit	Description
k_a	[1/h]	Absorption rate constant
CL	[mL/h]	Drug Clearance
V_c	[mL]	Volume of distribution
Q_1	[1/h]	Inter-compartmental clearance
V_{p1}	[mL]	Volume of distribution of the peripheral compartment
v_{max}	[1/h]	Maximum process rate
$V_{max,50}$	[ng/mL]	Drug concentration with half-maximum process rate
Variable		
A_d	[ng]	Amount of drug in dosing compartment (depot)
A_c	[ng]	Amount of drug in central compartment
A_{p1}	[ng]	Amount of drug in peripheral compartment

Table S7: Drug action models fitted for each drug and parasite growth model combination. The effect E was added to the parameter of parasite death α for *model a-h* and directly subtracted from the growth rate p_{gr} in case of *model i*. Bold parameters are calibrated against data during this modeling step.

**Model**	**Equation**	**Drug effect**
**Clearance**	$\frac{dY}{dt} = (\ldots) - \frac{\boldsymbol{E_{max}} C^{\gamma}}{\boldsymbol{EC_{50}^{\gamma}} + C^{\gamma}} Y$ $\frac{dY_{Cl}}{dt} = \frac{\boldsymbol{E_{max}} C^{\gamma}}{\boldsymbol{EC_{50}^{\gamma}} + C^{\gamma}} Y - \boldsymbol{Cl_Y} Y_{Cl}$	Parasites Y are damaged by the drug with direct effect, dead parasites Y_{Cl} are cleared with rate Cl_Y
**Effect**	$\frac{dC_e}{dt} = \boldsymbol{k_e}(C - C_e)$ $E = \frac{\boldsymbol{E_{max}} C_e^{\gamma}}{\boldsymbol{EC_{50}^{\gamma}} + C_e^{\gamma}}$	Reversible direct effect with additional effect compartment,<br>incorporates time-delay in drug action
**E_{max}**	$E = \frac{\boldsymbol{E_{max}} C^{\gamma}}{\boldsymbol{EC_{50}^{\gamma}} + C^{\gamma}}$	Direct effect of the drug
**Turnover**	$E = \boldsymbol{k_R} \boldsymbol{E_{max}} \left(\frac{C^{\gamma}}{\boldsymbol{EC_{50}^{\gamma}} + C^{\gamma}} - E \right)$	Indirect response

Parameter	Unit	Description
C	[mg/mL]	Drug concentration
C_e	[mg/mL]	Drug concentration in effect compartment
Cl_Y	[1/h]	Clearance rate for damaged parasites
E	[1/h]	Effect of the drug
EC_{50}	[mg/mL]	Drug concentration causing 50% of maximum effect
E_{max}	[1/h]	Maximum effect of the drug
k_e	[1/h]	First order rate constant for drug concentration in effect compartment
k_R	[1/h]	First order rate constant for biological intermediate
γ	[]	Hill-coefficient, parameter of steepness of the concentration-effect curve
Y_{Cl}	[cells/mL]	Parasites killed/damaged by the drug

Section 3: Parameter estimates

Table S8: Estimated parameter values of *P. berghei* and *P. falciparum* growth models. Parameter estimation was performed using data specified in Table S2 and equations noted in Table S3. Parameter ranges estimated over all experiments are given for parameters $\boldsymbol{\beta}$, $\boldsymbol{H_0}$, $\boldsymbol{P_0}$ and $\boldsymbol{p_{gr}}$.

Model	Description	Parameter (95% CI)	Unit	Error (prop, add [% parasitemia])
**Model a (base)**	β *	6.7e-11 – 1.7e-10	[cells/mLh]	0.35, 0.15
	r	14.5 (12.6;16.5)		
	ω	0.99 (0.99;0.99)		
**Model b (bystander)**	β *	1.94e-10 – 5e-10	[cells/mLh]	0.35, 0.15
	r	11.5 (10;13.2)		
	ω	0.28 (0.22;0.35)		
	y_{max}	0.02 (0.018;0.023)	[1/h]	
	ky_{50}	7.7e-5 (3.15e-5;1.9e-4)	[10^{10} cells/mL]	
**Model c (comp. erythr.)**	β *	7.1e-11 – 1.70e-10	[cells/mLh]	0.37, 0.15
	r	14.5 (12.7;16.5)		
	ω	0.99 (0.99;0.99)		
**Model d (impaired maturation)**	β *	9.8e-11 – 2.4e-10	[cells/mLh]	0.14, 0.14
	r	13.0 (11.4;14.9)		
	ω	0.74 (0.53;0.88)		
	$k_{l,50}$	0.12 (0.095;0.17)	[10^{10}cells/mL]	
**Model e (reticulocytes)**	β *	7.1e-11 – 1.2e-10	[cells/mLh]	0.39, 0.14
	r	10.6 (9.2;12.1)	[1/h]	
	ω	0.78 (0.12;0.99)		
	ε	5.1 (3.5;7.4)		
**Model f (const. RBC decay)**	β*	2.4e-10 – 1.6e-9	[cells/mLh]	0.31, 0.17
	H_0*	0.40 – 0.69		
	r	21.3 (18.6;24.5)		
	λ	0.010 (0.0099; 0.010)	[1/h]	
	γ_{max}	0.44 (0.38; 0.50)	[1/h]	
	$k\gamma_{50}$	0.24 (0.21; 0.27)	[cells/mL]	
	ω	0.29 (0.26; 0.33)		
	φ	0.030 (0.023; 0.0340)	[1/h]	
**Model g (dd. RBC decay)**	β*	2.0e-10 – 9.2e-10	[cells/mLh]	0.3, 0.001
	H_0*	0.40 – 0.65		
	r	22.8 (19.8;26.2)		
	ω	0.25 (0.18;0.34)		
	χ_{max}	0.018 (0.016;0.021)	[1/h]	
	$k\chi_{,50}$	1.05 (0.91;1.18)	[10^{10} cells/mL]	
	γ_{max}	0.055 (0.041;0.074)	[1/h]	
	$K\gamma_{50}$	0.10 (0.09;0.12)	[10^{10} cells/mL]	
	φ	0.027 (0.016;0.047)	[1/h]	
**Model h (human RBC)**	β*	2.1e-10 – 8.8e-10	[cells/mLh]	0.34, 0.00010
	H_0*	0.40 – 0.65		
	r	22 (21.7;22.3)		
	ω	0.36 (0.28;0.46)		
	γ_{max}	0.067 (0.046;0.098)	[1/h]	
	$k\gamma_{50}$	0.20 (0.17;0.24)	[10^{10} cells/mL]	
	λ	0.008 (0.008;0.008)	[1/h]	
	φ	0.040 (0.030;0.047)	[1/h]	
**Model i (exponential)**	P_0	-1.03 -1.52	[log(P)]	
	p_{gr}	0.16 – 0.30	[1/h]	

* range of values found over all experiments

Table S9: Estimates of the pharmacokinetic profile parameters for each investigated drug in infected mice. Estimates are given with their residual standard error [%]. Rates are given in [1/h], concentrations in [mg/mL] and volume in [mL]. Parameters were estimated using a NLME approach in Monolix (2016R1).

	**Drug**	**comp**	**Abs.**	**Elim**	**k_a [1/h]**	**CL [mL /h]**	**V_c [mL]**	**Q_1 [1/h]**	**V_{p1} [mL]**	**v_{max} [1/h]**	**$V_{max,50}$ [ng/mL]**	**Rel. err**
P. berghei **in normal mice**	ACT-451840	*2*	MM	lin.	-	51 (8)	91 (25)	1.09 (1.45e4)	0.08 (2.25e4)	0.89 (29)	5.47 (36)	0.31 (15)
	CQ	*2*	lin.	lin.	0.73 (23)	29 (5)	218 (12)	4.34 (47)	100 (24)			0.27 (11)
	MMV390048	*1*	lin.	MM, lin.	0.23 (16)	730 (13)	480 (21)			0.32 (21)	4.5 (121)	0.21 (11)
	OZ439	*2*	lin.	lin.	2.7 (63)	51 (14)	466 (19)	17 (211)	183 (534)			0.32 (25)
P. falciparim **in NOD scidIL-2R' c-/-**	ACT-451840	*1*	lin.	lin.	0.047 (14)	210 (11)	109 (47)					0.27 (24)
	CQ	*1*	lin.	lin.	26 (7480)	68 (32)	630 (30)					0.17 (17)
	MMV390048	*1*	lin.	MM, lin.	30 (7)	130 (4)	280 (33)			0.68 (5)	20 (22)	0.34 (4)
	OZ439	*2*	lin.	lin.	2.84 (19)	1.7 (-)	686 (9)	63 (5)	1.71e5 (34)			0.52 (5)

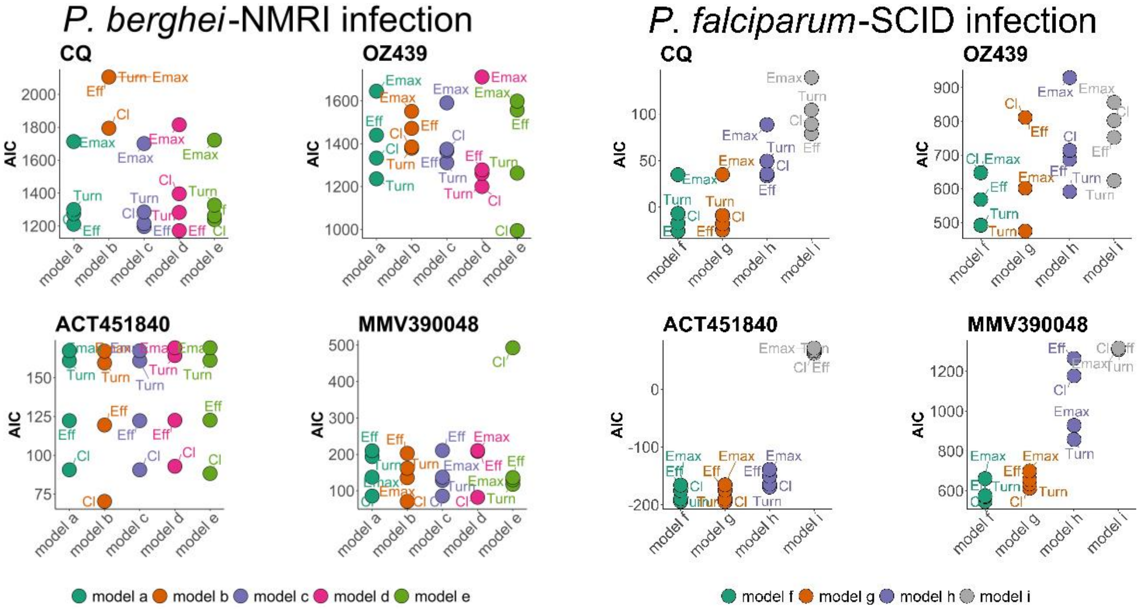

Figure S2: Selection of model of drug action based on AIC for the two murine experimental systems and four drugs. Models were selected based on lowest AIC and biological plausibility of the estimated parameters (E_{max}, EC_{50} and additional parameter). Models excluded due to biologically implausible parameters are set in parentheses.

Table S10: Estimated values of drug efficacy parameters. For each parasite growth model (PG), the drug action model with hill-coefficient *n* describing data best (ΔOFV) was chosen for comparison (see equations for drug action model in Table S6). Parameters are stated with their 95% confidence interval. Additional parameters are k_e for the effect model, k_R for the turnover model and Cl_Y for the clearance model.

	Drug	PG	Drug action	γ	**EC_{50}** [ng/mL] (95% CI)	**E_{max}** [1/h] (95% CI)	**Add. parameter**[1/h] (95 % CI)
***P. berghei* in normal mice**	ACT-451840	*a*	Clearance	5	1.1e2 (91;1.3 e2)	0.71 (0.41;1.2)	0.027 (0.025;0.029)
		b	Clearance	5	92 (82;1.0 e2)	0.50 (0.35;0.71)	0.026 (0.022;0.029)
		c	Clearance	5	1.1e2 (91;1.3 e2)	0.71 (0.41;1.2)	0.027 (0.025;0.029)
		d	Clearance	5	1e2 (85;1.2e2)	0.63 (0.41;0.97)	0.028 (0.026;0.031)
		e	Clearance	5	98 (85;1.1e2	0.60 (0.40;0.91)	0.032 (0.030;0.034)
	CQ	*a*	Effect	7	41 (39;43)	0.095 (0.093;0.097)	0.037 (0.036;0.038)
		b	Clearance	5	1.2e2 (98;1.4e2)	0.42 (0.35;0.50)	2.8e-3 (9.8e-5;4.8e-3)
		c	Effect	7	41 (39;43)	0.094 (0.092;0.096)	0.035 (0.034;0.037)
		d	Effect	2	46(43;50)	0.11 (0.11;0.11)	0.030 (0.028;0.031)
		e	Clearance	7	88 (83;94)	0.34 (0.33;0.35)	0.053 (0.051;0.055)
	MMV-390048	*a*	Clearance	5	3.7e2 (3.3e2;4.1e2)	0.61 (0.45;0.83)	0.039 (0.035;0.043)
		b	Clearance	7	2.8e2 (2.6e2;3.0e2)	0.39 (0.31;0.48)	0.036 (0.032;0.042)
		c	Clearance	5	3.7e2 (3.3e2;4.1e2)	0.61 (0.45;0.83;	0.039 (0.036;0.043)
		d	Clearance	5	3.1e2 (2.8e2;3.5e2)	0.45 (0.36;0.58)	0.041 (0.037;0.045)
		e	Turnover	2	2.2e2 (1.7e2;3.0e2)	0.19 (0.070;0.50)	0.055 (0.0054;0.55)
	OZ439	*a*	Turnover	7	49 (47;50)	0.93 (0.90;0.95)	0.013 (0.012;0.014)
		b	Turnover	7	49 (46;52)	0.94 (0.35;2.5)	0.013 (0.004;0.038)
		c	Turnover	7	42 (40;45)	0.28 (0.25;0.31)	0.060 (0.050;0.072)
		d	Turnover	7	46 (44;48)	0.84 (0.82;0.86)	0.015 (0.014;0.016)
		e	Turnover	7	46 (44;47)	0.91 (0.89;0.94)	0.014 (0.013;0.015)
***P. falciparim* in NOD scidIL-2R' c-/-**	ACT-451840	*f*	Turnover	5	17 (16;19)	0.093 (0.080;0.11)	0.062 (0.041;0.091)
		g	Turnover	5	17 (16;19)	0.095 (0.082; 0.11)	0.063 (0.062;0.073)
		h	Turnover	5	18 (17;19)	0.094 (0.082;0.11)	0.061 (0.041;0.088)
		i	Clearance	5	16 (15;18)	0.065 (0.062;0.067)	0.17 (0.14;0.19)
	CQ	*f*	Effect	7	71 (67;76)	0.098 (0.094;0.10)	0.044 (0.040;0.061)
		g	Effect	7	71 (67;75)	0.10 (0.095;0.10)	0.044 (0.038;0.059)
		h	Effect	7	70 (66;74)	0.098 (0.094;0.10)	0.045 (0.038;0.059)
		i	Effect	7	70 (66;75)	0.099 (0.092;0.11)	0.050 (0.042;0.060)
	MMV-390048	*f*	Clearance	2	1.2e2 (1.1e2;1.3e2)	0.090 (0.087;0.091)	0.071 (0.065;0.077)
		g	Clearance	5	1.1e2 (1.1 e2;1.2e2)	0.093 (0.091;0.096	0.068 (0.062;0.073)
		h	Turnover	7	1.1e2 (1.0e2;1.1e2)	0.082 (0.080;0.084)	0.073 (0.063;0.083)
		i	Clearance	5	32 (29;35)	0.12 (0.11;0.13)	0.025 (0.023;0.028)
	OZ439	*f*	Turnover	5	75 (71;79)	0.26 (0.25;0.26)	0.016 (0.015;0.017)
		g	Turnover	5	80 (76;85)	0.33 (0.32;0.34)	0.013 (0.012;0.014)
		h	Turnover	5	77 (73;82)	0.30 (0.30;0.31)	0.013 (0.013;0.014)
		i	Turnover	2	213 (197;230)	0.67 (0.63;0.72)	0.020 (0.018;0.023)

Section 4: Additional Findings

Table S11: Overview of experimental data of drug efficacy for *P. falciparum* infected NOD $^{scidIL\text{-}2R' \ c\text{-}/\text{-}}$ -mice treated with MMV390048 and OZ439. Only experiments conducted for sufficient duration parasites to recrudesce are listed. The total number of mice treated with the treatment regimen is split into mice cured and mice demonstrating recrudescence. For the remaining mice treatment did not decrease parasite numbers below the lower limit of quantification (0.01% parasitemia).

	Treatment regimen	no. mice	mice cured	mice with recrudescence	Time of recrudescence [hours after inoculation]
MMV390048	2×1 mg/kg	4	0	0	-
	2×10 mg/kg	5	0	2	336, 408
	4×0.25mg/kg	4	0	0	-
	4×0.5 mg/kg	8	0	0	-
	4×1 mg/kg	8	0	6	336, 456, 504, 504, 576, 624
	4×2.5 mg/kg	6	2	4	288, 576 ,576, 744
	4×5 mg/kg	2	2	0	-
	4×10 mg/kg	4	2	2	672,744
	4×20 mg/kg	2	2	0	-
OZ439	1×2.5 mg/kg	2	0	0	-
	1×5 mg/kg	2	0	0	-
	1×10 mg/kg	6	0	0	-
	1×20 mg/kg	2	0	2	288, 288
	1×30 mg/kg	2	0	2	336, 480
	1×50 mg/kg	2	0	2	576, 624
	1×75 mg/kg	2	0	2	408, 672
	1×100 mg/kg	2	2	0	-
	2×10 mg/kg	4	0	4	240, 240, 288, 336
	2×25 mg/kg	4	0	4	408, 456, 504, 672

Table S11: Individual slopes of parasite treatment curves and time of recrudescence for treatment with MMV390048 and OZ439 in SCID mice. The slopes of the parasite count curves after treatment were determined directly from the data.

**MMV390048**			**OZ439**		
**Time of recrudescence [hours after inoculation]**	**Dosing Regimen**	**-slope**	**Time of recrudescence [hours after inoculation]**	**Dosing Regimen**	**-slope**
288	4×2.5 mg/kg	0.0100	240	2×10 mg/kg	0.0030
336	4×1 mg/kg	0.0075	240	2×10 mg/kg	0.0021
336	2×10 mg/kg	0.0092	288	1×20 mg/kg	0.0281
408	2×10 mg/kg	0.0073	288	1×20 mg/kg	0.0264
456	4×1 mg/kg	0.0054	288	2×10 mg/kg	0.0020
504	4×1 mg/kg	0.0108	336	1×30 mg/kg	0.0256
504	4×1 mg/kg	0.0108	336	2×10 mg/kg	0.0061
576	4×1 mg/kg	0.0213	408	1×75 mg/kg	0.0174
576	4×2.5 mg/kg	0.0117	408	2×25 mg/kg	0.0085
576	4×2.5 mg/kg	0.0117	456	2×25 mg/kg	0.0058
624	4×1 mg/kg	0.0113	480	1×30 mg/kg	0.0281
672	4×10 mg/kg	0.0088	504	2×25 mg/kg	0.0071
744	4×2.5 mg/kg	0.0129	576	1×50 mg/kg	0.0291
744	4×10 mg/kg	0.0091	624	1×50 mg/kg	0.0337
			672	1×75 mg/kg	0.0296
			672	2×25 mg/kg	0.0080

Table S12: Regression analysis for slope of parasite treatment curve and recrudescence times. Ordinary linear regression (least-squares) was conducted to analyze the correlation between time of recrudescence (y) for each individual mouse and the slope of the parasite concentration curve after treatment calculated from individual mouse data (x_1, b_1), number of doses (x_2, b_2), and dosing amount (x_3, b_3) using the regression equation y= b_1 x+ b_2 x + b_3 x +b_0.We tested all combinations of predictors. The data used for the analysis can be found in Table S10 and Table S11.

	Slope b_1 (p-value)	Regimen b_2 (p-value)	Dose b_3 (p-value)	Regression p-value	R^2
**MMV390048**	13440 (0.240)	-	-	0.240	0.0392
	-	-	3.3 (0.761)	0.761	-0.07
	-	89 (0.116)	-	0.165	0.125
	9434 (0.404)	76 (0.194)	-	0.214	0.107
	16486 (0.187)	-	8.4 (0.454)	0.386	0.00625
	-	169 (0.0171)	23 (0.0608)	0.0492	0.317
	14558 (0.142)	163 (0.167)	27 (0.0300)	0.0442	0.401
**OZ439**	4936 (0.133)	-	-	0.133	0.0932
	-	-	4.5 (0.008)	0.00780	0.363
	-	-66 (0.396)	0	0.396	-0.01
	-	169 (0.00793)	23 (0.00793)	0.0183	0.377
	23486 (0.0215)	448 (0.0493)	-	0.0455	0.283
	249 (0.942)	-	4.4 (0.0359)	0.0340	0.314
	25009 (0.000267)	645 (0.000148)	6.2 (0.000112)	6.80e-5	0.786

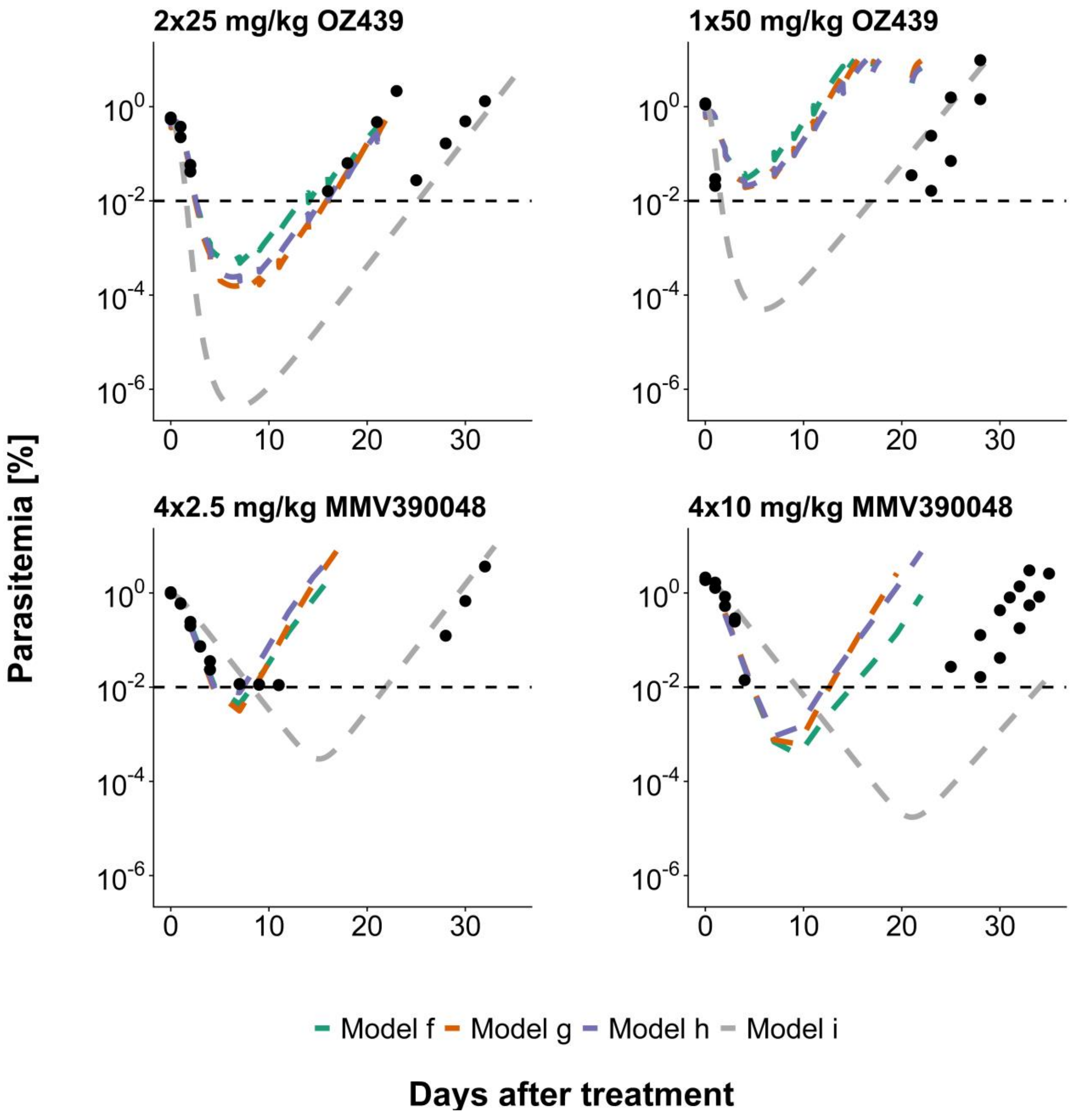

Figure S3: Typical fit of drug action models of SCID mice infected with *P. falciparum* demonstrating late recrudescence. Infection occurred at day 0 with an inoculum of 2×107-3.5×107 infected RBCs. Treatment commenced three days after inoculation in dosing intervals of 24 hours. Mice were treated with 2×25 mg/kg OZ439, 1×50 mg/kg OZ439, 4×2.5 mg/kg MMV390048 or 4×10 mg/kg MMV390048. n=2 mice for all doses shown.

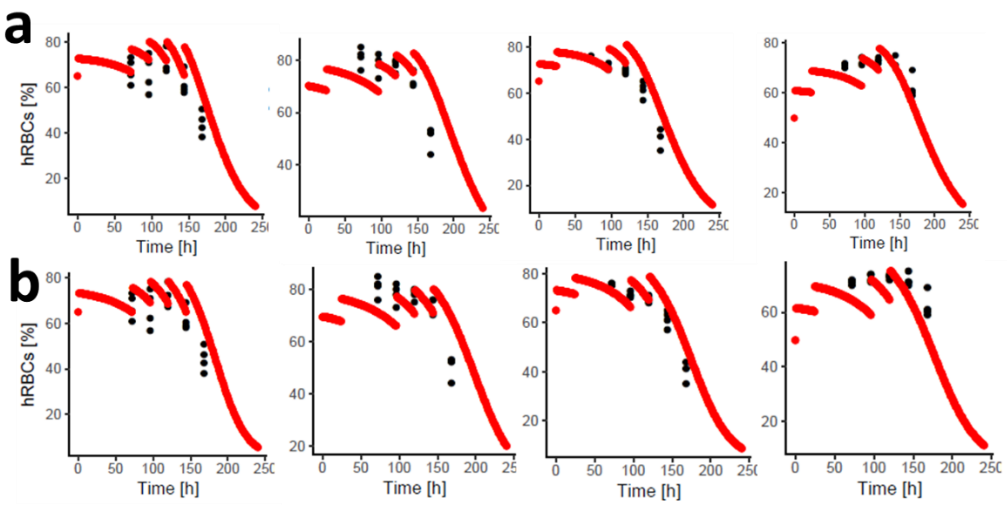

Figure S4: **The influence of ceasing human erythrocyte injections in SCID mice infected with *P. falciparum*.** Data (black •) and model output (red line) of four experiments, with human RBC injections ceasing before data collection, is shown for (a) *model f (const. RBC decay)* and (b) *model g (dd. RBC decay)*. RBC injections are indicated by sudden increase in hRBCs in the model output.

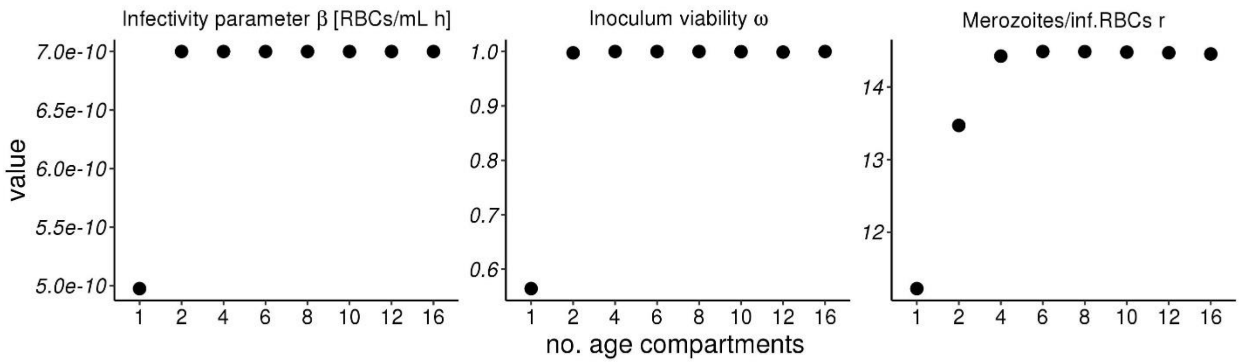

Figure S5: **Stability analysis conducted for the base structure of the models (corresponding to *model a (base)* towards the number of intra-erythrocytic parasite age-stages.** Total values of parameter estimates for *model a (base)* including n=1,2..,16 age compartments show the most prominent influence of the number of age compartments between one and two compartments with no distinctive contribution of additional age compartments. Considering the stability analysis and computational efficacy, we chose n=12 splitting the parasite life cycle in time-steps of two hours for *P.berghei* and four hours for *P.falciparum* considering the stability analysis and computational efficacy.

References

1 Boss, C. *et al.* Discovery and characterization of ACT-451840: an antimalarial drug with a novel mechanism of action. *ChemMedChem* **11**, 1995-2014, doi:10.1002/cmdc.201600298 (2016).

2 Le Bihan, A. *et al.* Characterization of novel antimalarial compound ACT-451840: preclinical assessment of activity and dose-efficacy modeling. *PLoS medicine* **13**, e1002138, doi:10.1371/journal.pmed.1002138 (2016).

3 Phillips, M. A. *et al.* Malaria. *Nature Reviews Disease Primers* **3**, 17050, doi:10.1038/nrdp.2017.50 (2017).

4 Paquet, T. *et al.* Antimalarial efficacy of MMV390048, an inhibitor of Plasmodium phosphatidylinositol 4-kinase. *Science translational medicine* **9**, doi:10.1126/scitranslmed.aad9735 (2017).

5 Dong, Y. *et al.* Structure-activity relationship of the antimalarial ozonide artefenomel (OZ439). *J Med Chem* **60**, 2654-2668, doi:10.1021/acs.jmedchem.6b01586 (2017).

6 Charman, S. A. *et al.* Synthetic ozonide drug candidate OZ439 offers new hope for a single-dose cure of uncomplicated malaria. *Proceedings of the National Academy of Sciences of the United States of America* **108**, 4400-4405, doi:10.1073/pnas.1015762108 (2011).

7 Hetzel, C. & Anderson, R. M. The within-host cellular dynamics of bloodstage malaria: theoretical and experimental studies. *Parasitology* **113**, 25-38 (1996).

8 Cromer, D., Evans, K. J., Schofield, L. & Davenport, M. P. Preferential invasion of reticulocytes during late-stage *Plasmodium berghei* infection accounts for reduced circulating reticulocyte levels. *International Journal for Parasitology* **36**, 1389-1397, doi:https://doi.org/10.1016/j.ijpara.2006.07.009 (2006).

9 Angulo-Barturen, I. *et al.* A murine model of falciparum-malaria by in vivo selection of competent strains in non-myelodepleted mice engrafted with human erythrocytes. *PLoS One* **3**, e2252, doi:10.1371/journal.pone.0002252 (2008).

10 Lloyd, S. W. M. in *Handbook of Laboratory Animal Management and Welfare* Vol. 3 Ch. 9, (Blackwell Publishing, 2003).
